# Heterogeneous Mediation Analysis for Cox Proportional Hazards Model With Multiple Mediators

**DOI:** 10.1002/sim.10239

**Published:** 2024-10-28

**Authors:** Rongqian Sun, Xinyuan Song

**Affiliations:** ^1^ School of Psychology Shenzhen University Shenzhen China; ^2^ Department of Statistics Chinese University of Hong Kong Hong Kong China

**Keywords:** heterogeneous effect, multiple mediators, survival outcome, variable selection

## Abstract

This study proposes a heterogeneous mediation analysis for survival data that accommodates multiple mediators and sparsity of the predictors. We introduce a joint modeling approach that links the mediation regression and proportional hazards models through Bayesian additive regression trees with shared typologies. The shared tree component is motivated by the fact that confounders and effect modifiers on the causal pathways linked by different mediators often overlap. A sparsity‐inducing prior is incorporated to capture the most relevant confounders and effect modifiers on different causal pathways. The individual‐specific interventional direct and indirect effects are derived on the scale of the logarithm of hazards and survival function. A Bayesian approach with an efficient Markov chain Monte Carlo algorithm is developed to estimate the conditional interventional effects through the Monte Carlo implementation of the mediation formula. Simulation studies are conducted to verify the empirical performance of the proposed method. An application to the ACTG175 study further demonstrates the method's utility in causal discovery and heterogeneity quantification.

## 1 Introduction

Unraveling the mechanisms through which an exposure or treatment exerts its effect on an outcome of interest is a fundamental pursuit across multiple disciplines, including epidemiology, psychology, and social sciences. Mediation analysis has emerged as a powerful tool for disentangling the intricate causal pathways from the exposure to the outcome, which can be implemented through one or more intermediate variables. Various mediation models have been developed in survival analysis to elucidate the causal mechanism underlying the relationship between a therapy or risk factor and a time‐to‐event outcome. A majority of these approaches are grounded in classic frameworks, such as a linear structural equation model [1] combined with an additive hazards model [2, 3], proportional hazards model [4, 5], or transformation models [6, 7], enabling efficient estimation of the population‐level direct and indirect effects with good interpretability.

Beyond the scope of mediation analysis, the growing accessibility of large‐scale clinical trials and observational studies, coupled with recent advancements in machine learning techniques, has inevitably shifted the focus of causal inference from population‐level average treatment effects (ATE) to group‐level conditional average treatment effects (CATE) given individual‐specific features. This trend is prominent in biomedical studies involving survival data, where patients with distinct characteristics naturally respond differently to the same treatment. The rise of precision medicine has fueled the development of various approaches for estimating heterogeneous treatment effects, including causal survival forest [8], nonparametric accelerated failure time model based on Bayesian trees [9, 10], subgroup analysis via gradient tree boosting [11], and deep neural networks‐based Cox regression [12]. However, these approaches primarily focus on capturing heterogeneity within the total effect of the exposure on the survival outcome. They often overlook the potential existence of embedded causal pathways, let alone the more intricate scenario where multiple mediators are present, each with the possibility of heterogeneity occurrence. Although conventional moderated mediation analysis and its recent adaptation to the causal framework [13, 14] provide a straightforward solution by including first‐order interaction terms of a suspected moderator with the exposure in the mediator(s) regression model, as well as the interaction of the suspected moderator with the exposure or the mediator(s) in the outcome regression model, it typically targets on one moderator at a time and can become less practical when abundant choices of moderators exist.

On the other hand, large‐scale trials and observational datasets often come with many pretreatment covariates or even posttreatment covariates serving as potential mediators. However, a substantial proportion of these covariates may neither affect the mediator/outcome nor interact with the exposure. In other words, in practical applications, it is often the case that only a small portion of the pretreatment covariates truly interact with the exposure or the mediators on their pathways to the outcome, and similarly, only a small part of the posttreatment variables genuinely mediate the effect of the exposure on the outcome. In such cases, the accurate estimation of CATE for different subgroups and the reliability of the detected sources of heterogeneity inevitably rely on feature selection for the sparse regression surface. Therefore, it is critically important to differentiate the genuine confounders and effect modifiers from the irrelevant covariates and, more crucially, to distinguish the true mediators from a set of posttreatment covariates that were measured temporally between treatment initiation and the outcome of interest, and consequently included among the candidate mediators. Although recent literature has seen various works addressing this issue for causal survival analysis [15, 16] or high‐dimensional mediation analysis [17], these approaches have yet to consider a simultaneous selection of confounders, effect modifiers, and mediators for heterogeneous mediation analysis with a survival outcome.

To address these challenges, we propose a novel Bayesian semiparametric approach for mediation analysis with survival outcomes that adapts to the existence of multiple mediators, heterogeneity across causal pathways, sparsity of the regression surfaces, and overlapping patterns of confounders or effect modifiers across different pathways. Our model comprises two major components built upon the Bayesian Causal Forest (BCF) [18] framework, which provides a flexible and interpretable way to estimate CATE. Specifically, the first component is a mediator regression model, which characterizes the prognostic factors of the exposure–mediator relationships and modifiers of the direct effect on the mediators through two separate Bayesian additive regression trees (BART) [19]. Motivated by the mixed‐scale Bayesian forest [20], we allow the tree typologies within each BART to be shared among the mediators, such that the overlap of the confounders or effect modifiers can be accommodated straightforwardly. The second component fits the survival outcome through a proportional hazards (PH) model with two separate BART, one capturing the prognostic factors of the exposure–outcome relationship and the other describing the direct effect of the exposure on the logarithm of hazards. The PH model is linked to the mediator regression model in that the second BART is allowed to share tree typologies with its counterpart in the mediator regression model, thereby accommodating the presence of overlapping modifiers for the direct effects of the exposure. Besides, we extend the BCF to incorporate shrinkage priors for variable selection [21], enabling the selection of relevant confounders and effect modifiers while simultaneously fitting the possibly complex and nonlinear regression surfaces. By combining the flexibility of a Bayesian nonparametric ensemble of trees with shared typologies and sparsity‐inducing prior, the proposed approach offers a powerful tool for uncovering heterogeneous mediation mechanisms in survival data without imposing restrictive assumptions on the regression surfaces or requiring manual variable preselection.

The rest of the article is organized as follows. Section 2 introduces the proposed heterogeneous mediation model with shared tree ensembles. Section 3 defines the interventional path‐specific effects under the potential outcomes framework, along with a set of identifiability assumptions. Section 4 elucidates the Bayesian inference procedure for the proposed methodology. Section 5 presents simulation studies that evaluate the empirical performance of the proposed model. Section 6 provides a real‐world application example using a dataset collected from a clinical trial on HIV‐infected patients. Section 7 concludes the paper. Technical details are provided in the Data S1.

## 2 Model Description

### 2.1 A Brief Overview of BART

Let $x={\left(x_{1},\ldots ,x_{p}\right)}^{T}$ be a p×1 vector of predictors and Y be a continuous response variable. BART is a flexible nonparametric model that approximates the unknown regression function of Y on x via a sum of J binary trees, where each binary tree can be viewed as a simple step function that splits the original dataset into more homogeneous subsets according to the values of x. Such a regression problem is usually set up as

(1) $$Y=f(x)+\epsilon =\overset{J}{\underset{j=1}{\sum }}g(x;{𝒯}_{j},{ℳ}_{j})+\epsilon ,\;\epsilon ∼N(0,\sigma^{2})$$

where f(x) is the true unknown regression function to be learnt from the data and ϵ is a normally distributed error term with zero mean. ${𝒯}_{j}$ denotes the structure of the jth binary tree composed of a collection of internal nodes with splitting rules and $b_{j}$ terminal nodes, and ${ℳ}_{j}={\left(\mu_{j1},\ldots ,\mu_{jb_{j}}\right)}^{T}$ denotes the vector of parameter values corresponding to each terminal node, with $\mu_{jl}$ serving as a gauge of the average outcome inside the lth node. At each internal node, binary splits are made according to rules of the form $\left\{x_{s}\leq c\right\}$ vs. $\left\{x_{s}>c\right\}$, with $x_{s}\;(s\in \{1,\ldots ,p\})$ being the sth predictor and c being a threshold. The top‐down sequence of all such splitting rules across each binary tree ${𝒯}_{j}$, as a whole, recursively partitions the original covariate space ${\mathbb{R}}^{p}$ into $b_{j}$ subsets represented by the terminal nodes. $g(x;{𝒯}_{j},{ℳ}_{j})$ is a function that maps a given x to a node parameter $\mu_{jl}\in {ℳ}_{j}$ suppose that it is allocated to the lth ($l=1,\ldots ,b_{j}$) terminal node of ${𝒯}_{j}$ according to the above rules, thereby ending up as a piecewise constant function. Since any given x is placed to one unique terminal node within each tree, f(x) (or the conditional mean function E(Y|x)) is fitted as a sum of the corresponding $\mu_{jl}$s over the J trees.

The BART framework has seen significant extensions to handle diverse data types, including mixed‐scale responses, time‐to‐event outcomes, and high‐dimensional predictors. Notable examples include the BCF, which introduced tailored regularization for estimating CATEs, and shrinkage priors [21, 22, 23] that enable variable selection from redundant covariates, among others. In the next section, we will build upon the BCF framework to introduce the proposed heterogeneous mediation model. For a comprehensive introduction to BART, readers are directed to Tan and Roy [24], while Hill, Linero, and Murray [25] provide an overview of recent developments in this area.

### 2.2 Parallel Mediators With Shared Ensembles

We consider a two‐arm study design with $p_{m}$ continuous mediator(s) and a right‐censored time‐to‐event outcome. For subject i=1,…,n, let $x_{i}$ denote the vector of pretreatment covariates. Let $A_{i}\in \{0,1\}$ be the treatment indicator, with $A_{i}=1$ if subject i is assigned to the treated group and $A_{i}=0$ otherwise. Let $M_{i}=(M_{i1},\ldots ,M_{i,p_{m}})$ be a $p_{m}\times 1$ vector of parallel (i.e., causally non‐ordered) mediators with each element representing a specific mediator that links a potential causal pathway between the treatment and the time‐to‐event outcome. Causal heterogeneity is usually characterized as interaction between the treatment indicator $A_{i}$ and a subset of $x_{i}$, also known as the effect modifiers. Such effect modifiers in moderated mediation analysis can be shared across pathways from the treatment to multiple mediators. Therefore, we model the parallel mediators $M_{i}$ through two shared ensembles of trees as follows:

(2) $$M_{i}=\upsilon_{M}(x_{i})+\tau_{M}(x_{i})A_{i}+\epsilon_{i}$$

where $\upsilon_{M}(x_{i})=\sum_{j=1}^{J}g({𝒯}_{j},{ℳ}_{j})$ is a $p_{m}\times 1$ dimensional vector that captures the prognostic factors of the treatment‐mediator relationship through the ensemble of J binary trees ${\left\{{𝒯}_{j}\right\}}_{j=1}^{J}$ with leaf node parameters ${\left\{{ℳ}_{j}={\left(\mu_{j1},\ldots ,\mu_{jb_{j}}\right)}^{T}\right\}}_{j=1}^{J}$ and $\mu_{jl}={\left(\mu_{jl,1},\ldots ,\mu_{jl,p_{m}}\right)}^{T}$, $l=1,\ldots ,b_{j}$. $\tau_{M}(x_{i})=\sum_{h=1}^{H}g({\tilde{𝒯}}_{h},{\tilde{ℳ}}_{h}^{\left(M\right)})$ is another BART that captures the direct effect of the treatment on the mediators through shared tree topology ${\left\{{\tilde{𝒯}}_{h}\right\}}_{h=1}^{H}$ with leaf node parameters ${\left\{{\tilde{ℳ}}_{h}^{\left(M\right)}={\left({\tilde{\mu }}_{h1}^{\left(M\right)},\ldots ,{\tilde{\mu }}_{hb_{h}}^{\left(M\right)}\right)}^{T}\right\}}_{h=1}^{H}$ and ${\tilde{\mu }}_{hl}^{\left(M\right)}={\left({\tilde{\mu }}_{hl,1}^{\left(M\right)},\ldots ,{\tilde{\mu }}_{hl,p_{m}}^{\left(M\right)}\right)}^{T}$. Note that $\mu_{jl}$ and ${\tilde{\mu }}_{hl}^{\left(M\right)}$ within ${𝒯}_{j}$ and ${\tilde{𝒯}}_{h}$ are $p_{m}\times 1$ dimensional vectors since the mediators are modeled jointly without any prespecified causal structure. For a special single‐mediator case with $p_{m}=1$, $\upsilon_{M}(x_{i})$ and $\tau_{M}(x_{i})$ in Equation (2) simply degenerate to two regular BART (i.e., $\upsilon_{M}(x_{i})$ and $\tau_{M}(x_{i})$) with one‐dimensional leaf node parameters $\mu_{jl}$ and ${\tilde{\mu }}_{hl}^{\left(M\right)}$, respectively. g(·) denotes the corresponding node‐parameter‐allocation functions. $\epsilon_{i}\overset{i.i.d}{∼}N(0,\Sigma )$ is the residual term of the mediator regression equations. While an alternative approach is to model the residual distribution nonparametrically as a location mixture of Gaussian distributions (see, e.g., Henderson et al. [9]), we found through a pilot simulation that combining the shared tree ensembles and Dirichlet process prior did not synergistically improve estimation accuracy of the proposed method. The additional model complexity introduced rendered it less desirable compared to the simpler assumption of normally distributed residuals.

Our model development up to this point has focused on scenarios where the mediators are continuous variables. However, it is straightforward to extend the mediator regression model to accommodate binary mediators using the multivariate probit model. Specifically,

(3) $$\begin{array}{ll} M_{i}^{*} & =\upsilon_{M}(x_{i})+\tau_{M}(x_{i})A_{i}+\epsilon_{i}\\ M_{i} & ={\left(I(M_{i1}^{*}>0),\ldots ,I(M_{i,p_{m}}^{*}>0)\right)}^{T}=I(M_{i}^{*}>0) \end{array}$$

where $M_{i}={\left(M_{i1},\ldots ,M_{i,p_{m}}\right)}^{T}\in {\left\{0,1\right\}}^{p_{m}}$ are the observed binary mediators, $M_{i}^{⋆}={\left(M_{i1}^{*},\ldots ,M_{i,p_{m}}^{*}\right)}^{T}$ are the underlying latent Gaussian variables as introduced by Chib and Greenberg [26], I(·) is the indicator function, and $\epsilon_{i}\overset{i.i.d}{∼}N(0,\Sigma )$ are the residuals with the diagonal elements of Σ fixed at 1.0. Similar extensions to accommodate binary mediators or outcomes have been adopted and seen to be effective in existing BART‐based causal models [24, 25, 27].

### 2.3 Cox Proportional Hazards Model

Let $T_{i}$ and $C_{i}$ represent the event time of interest and censoring time for subject i, respectively. Define $\delta_{i}=I(T_{i}<C_{i})$ as an indicator of whether the ith subject experiences the event of interest or is censored, and $Y_{i}=\min(T_{i},C_{i})$ as the observed time. Throughout this paper, we consider the scenario where a subject has the event or is censored after the mediators have been measured. Recognizing that effect modifiers can exert influence on multiple causal pathways simultaneously, that is, from both the treatment to the mediators and the treatment to the outcome, we employ the shared tree ensembles again in the PH model to jointly capture the causal relationships between the treatment, mediators, and the hazard at time t:

(4) $$\lambda (t|x_{i},M_{i},A_{i})=\lambda_{0}(t)\exp\{\upsilon (x_{i},M_{i})+\tau (x_{i})A_{i}\}$$

where $\tau (x_{i})=\sum_{h=1}^{H}g({\tilde{𝒯}}_{h},{\tilde{ℳ}}_{h})$ captures the direct effect of the treatment on hazard through shared the tree topology ${\left\{{\tilde{𝒯}}_{h}\right\}}_{h=1}^{H}$ with $\tau_{M}(x_{i})$ but possesses a different set of leaf node parameters, ${\left\{{\tilde{ℳ}}_{h}={\left({\tilde{\mu }}_{h1},\ldots ,{\tilde{\mu }}_{hb_{h}}\right)}^{T}\right\}}_{h=1}^{H}$. $\upsilon (x_{i},M_{i})=\sum_{k=1}^{K}g({\overset{˘}{𝒯}}_{k},{\overset{˘}{ℳ}}_{k})$ is another separate BART that quantifies the prognostic factors, or equivalently, the main effects and interaction of the pretreatment covariates and the mediators, on the pathways to the survival response through the sum of K binary trees ${\left\{{\overset{˘}{𝒯}}_{k}\right\}}_{k=1}^{K}$ with leaf node parameters ${\left\{{\overset{˘}{ℳ}}_{k}={\left({\overset{˘}{\mu }}_{k1},\ldots ,{\overset{˘}{\mu }}_{kb_{k}}\right)}^{T}\right\}}_{k=1}^{K}$. $\lambda_{0}(t)$ is the unknown baseline hazard function.

The proposed modeling approach stems from the frequent occurrence of shared effect modifiers influencing both the treatment–mediator and treatment–outcome relationships in real‐world scenarios. These shared modifiers can exhibit consistent moderation patterns, even in high‐dimensional settings with a sparse subset of true modifiers. The prognostic factors in the treatment–mediator relationship may also demonstrate an overlapped pattern. The shared tree typologies, ${\left\{{𝒯}_{j}\right\}}_{j=1}^{J}$ and ${\left\{{\tilde{𝒯}}_{h}\right\}}_{h=1}^{H}$, naturally accommodate the overlapped prognostic factors or moderators in the causal pathways. Besides, our approach extends moderated mediation to a semiparametric framework, accommodating arbitrary interactions among the effect modifiers and the treatment. This flexibility applies to both the mediator regression model and the outcome regression model, in contrast to the classical use of first‐order interaction terms. The BCF structure on the right‐hand side of Equations ((2), (3), (4)) directly captures the confounders and modifiers as splitting variables within different tree ensembles, with no need to prespecify the functional form of the covariates.

### 2.4 Model Specification and Variable Selection

We adopt the default BART prior outlined in Chipman, George, and McCulloch [19] and the BCF regularization prior of Hahn, Murray, and Carvalho [18] to specify the proposed model. However, we introduce a slight modification to account for the shared tree structure inherent in our modeling approach. For the mediator regression equation, consider first the following priors on each binary tree in ${\left\{{𝒯}_{j},{ℳ}_{j}\right\}}_{j=1}^{J}$ or ${\left\{{\tilde{𝒯}}_{h},{\tilde{ℳ}}_{h}^{\left(M\right)}\right\}}_{h=1}^{H}$: (i) the probability that a node at depth d(d∈{0,1,2,…}) continues splitting is given by $\alpha {\left(1+d\right)}^{-\beta }$ or $\tilde{\alpha }{\left(1+d\right)}^{\left(-\tilde{\beta }\right)}$, where $\alpha ,\tilde{\alpha }\in (0,1)$ and $\beta ,\tilde{\beta }>0$ are hyperparameters set a priori to control the scope of each binary tree; (ii) the splitting variable at each internal node is selected uniformly from the set of all available variables, and the splitting value at the internal node given the known splitting variable is drawn uniformly from the set of all available splitting values constructed from the interpolated sample quantiles; and (iii) given ${𝒯}_{j}$ or ${\tilde{𝒯}}_{h}$, the leaf node parameters $\mu_{jl}\in {ℳ}_{j}$ or ${\tilde{\mu }}_{hl}^{\left(M\right)}\in {\tilde{ℳ}}_{h}^{\left(M\right)}$ are assumed with conjugate normal priors $N\left(0,\frac{c_{1}^{2}}{4Jk_{1}^{2}}\Sigma_{0}\right)$ or $N\left(0,\frac{c_{2}^{2}}{4Hk_{2}^{2}}{\tilde{\Sigma }}_{0}\right)$, respectively, where $\left\{c_{1},c_{2},k_{1},k_{2}\right\}$ are prespecified hyperparameters that ensure a substantial prior probability is assigned within a desirable range for each mediator marginally, and $\Sigma_{0}$ and ${\tilde{\Sigma }}_{0}$ are $p_{m}\times p_{m}$ diagonal positive definite matrices. Priors (i)–(iii) collectively impose a regularization effect on each individual binary tree, restricting it to a weak learner that contributes only a small portion to the overall fit. The sequential accumulation of such weak learners under such regularization helps mitigate the risk of overfitting.

A common concern regarding the default BART prior is its potential limitation in high‐dimensional settings with a substantial number of irrelevant predictors. The discrete uniform prior (ii) used for predictor selection lacks an explicit mechanism for inducing sparsity or feature shrinkage. Specifically, consider $s=(s_{1},\ldots ,s_{p})$ as the vector of splitting probabilities for the predictors $x_{1},\ldots ,x_{p}$. The discrete uniform prior with $s_{j}=1/p$ for j∈{1,…,p} gives each predictor an equal chance of being selected as a splitting variable. Consequently, it may struggle to identify the real confounders or effect modifiers in our mediation problem, hindering model accuracy and interpretability. As an alternative, we employ the Gibbs‐type prior proposed by Linero and Du [21] to achieve variable selection in the proposed heterogeneous mediation analysis. This sparsity‐inducing prior is given as follows:

(5) (ii′):Z∼ϖZ,ϖZ(d)=d−ϰ/∑j=1pj−ϰ [𝒮|Z]∼pZ−1I(|𝒮|=Z) [s|𝒮]∼Dirichlet(αDI(1∈𝒮),…,αDI(p∈𝒮))

For an internal node at depth d, the idea is to first sample Z, the number of predictors allowed to be split on. A subset of predictors, 𝒮 of size Z, is then sampled from the complete set of predictors $\left\{x_{j},j=1,\ldots ,p\right\}$. For the sampled predictors with j∈𝒮, a Dirichlet prior is assigned to the corresponding splitting probability $s_{j}$. ϰ>0 is a hyperparameter that encodes the preference for sparsity. Notably, with ϰ=0 and $\alpha_{D}=1$, the Gibbs‐type prior (ii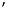) reduces to the discrete uniform prior.

We use a similar approach to specify the ensemble of trees in the PH model, assuming a prior node‐splitting probability of $\overset{˘}{\alpha }{\left(1+d\right)}^{-\overset{˘}{\beta }}$ for each binary tree in ${\left\{{\overset{˘}{𝒯}}_{k}\right\}}_{k=1}^{K}$ and the same sparsity‐inducing prior for the splitting rules as described in (ii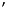) above. Throughout the paper, we consider a scenario where the dimension of the candidate mediators, $p_{m}$, do not necessarily grow as the number of observations increases and remains relatively small (as demonstrated in Sections 5 and 6). The primary difference lies in priors assigned to the leaf node parameters, ${\overset{˘}{\mu }}_{kl}\in {\overset{˘}{ℳ}}_{k}$ and ${\tilde{\mu }}_{hl}\in {\tilde{ℳ}}_{h}$. Based on the Bayesian justification of Cox's partial likelihood [28], we assign conjugate log‐gamma priors to the leaf node parameters, that is, ${\overset{˘}{\mu }}_{kl}∼\text{logGam}(\overset{˘}{\zeta },\overset{˘}{\eta })$ and ${\tilde{\mu }}_{hl}∼\text{logGam}(\tilde{\zeta },\tilde{\eta })$, where $\left\{\overset{˘}{\zeta },\overset{˘}{\eta },\tilde{\zeta },\tilde{\eta }\right\}$ are hyperparameters that ensure each binary tree a weak learner and a reasonable prior range for the ensemble. Section 4 contains a detailed description on choice of the hyperparameters.

## 3 Definition and Identification of the Interventional Conditional Effects

In this section, we construct the interventional conditional path‐specific effects (ICPSEs) under the counterfactual framework, using “distribution shifts” of the potential outcomes given the covariates. Let $T_{i}(a,m_{1},\ldots ,m_{p_{m}})$ be the potential survival time that would have been observed if the ith subject had been exposed to a treatment level of A=a and a mediator level of $M={\left(m_{1},\ldots ,m_{p_{m}}\right)}^{T}$, where each element $M_{iq}$ is set to the level of $m_{q}$, $q=1,\ldots ,p_{m}$. Let $M_{iq}(a_{q})$ be the qth potential mediator that would have been observed for the ith subject had the treatment level been set to $A=a_{q}$. Following the definition of interventional (in)direct effects [29, 30], we further denote $T_{i}(a,{\overset{'}{M}}_{i1}(a_{1}),\ldots ,{\overset{'}{M}}_{i,p_{m}}(a_{p_{m}}))$ as the potential survival time that would have been observed for the ith subject if the treatment level is set to A=a and the value of each mediator $M_{iq}$ is set to a random draw from its (counterfactual) marginal distribuion under a treatment level $a_{q}$, that is, ${\overset{'}{M}}_{iq}(a_{q})∼F_{M_{iq}(a_{q})|x_{i}}(m_{q})$, with $a,a_{q}\in \{0,1\}$, $q=1,\ldots ,p_{m}$, and $F_{B|C}$ denoting the cumulative distribution function of B conditional on C. Comparatively, let $T_{i}(a,\{{\overset{'}{M}}_{i1}(a^{\prime }),\ldots ,{\overset{'}{M}}_{i,p_{m}}(a^{\prime })\})$ be the potential survival time for the ith subject if the treatment level is set to A=a and the mediator levels are set to a random draw from the (counterfactual) joint distribution of the potential mediators under a treatment level $a^{\prime }$, i.e., $\left\{{\overset{'}{M}}_{i1}(a^{\prime }),\ldots ,{\overset{'}{M}}_{iq}(a^{\prime })\}∼F_{M_{i1}(a^{\prime }),\ldots ,M_{ip_{m}}(a^{\prime })|x_{i}}(m_{1},\ldots ,m_{p_{m}}\right)$, with $a,a^{\prime }\in \{0,1\}$.

Throughout this paper, we invoke three widely accepted assumptions that underpin causal inference: the stable unit treatment value assumption (SUTVA), the positivity assumption, and the consistency assumption. SUTVA postulates that treatment assignment for one individual does not influence the potential outcomes of any other individual, ruling out interference between units. The positivity assumption ensures that all units have a nonzero probability of being assigned to any of the treatment arms, preventing scenarios where certain subgroups are deterministically excluded from a specific treatment level. Within each treatment arm, it is further stipulated that densities of the candidate mediators conditional on the pretreatment covariates are nonzero with probability 1 for each value of their respective support. The consistency assumption states that an individual's potential mediators or potential outcome under the treatment condition they actually experienced is precisely their observed mediators/outcome.

Let $\omega \left\{T_{i}(a,m_{1},\ldots ,m_{p_{m}})|x_{i}\right\}$ denote a user‐specified function of the potential survival time conditional on the covariates. We define the conditional path‐specific effects under the interventional framework by substituting $T_{i}(a,m_{1},\ldots ,m_{p_{m}})$ with $T_{i}(a,{\overset{'}{M}}_{i1}(a_{1}),\ldots ,{\overset{'}{M}}_{i,p_{m}}(a_{p_{m}}))$ or $T_{i}(a,\{{\overset{'}{M}}_{i1}(a^{\prime }),\ldots ,{\overset{'}{M}}_{i,p_{m}}(a^{\prime })\})$. For notation simplicity, we omit $x_{i}$ and write it as $\omega \left\{T_{i}(a,{\overset{'}{M}}_{i1}(a_{1}),\ldots ,{\overset{'}{M}}_{i,p_{m}}(a_{p_{m}}))\right\}$ or $\omega \left\{T_{i}(a,\{{\overset{'}{M}}_{i1}(a^{\prime }),\ldots ,{\overset{'}{M}}_{i,p_{m}}(a^{\prime })\})\right\}$ in what follows. For each individual, the conditional average total effect of the binary treatment $A_{i}$ is defined as

(6) $$\begin{array}{ll} \Omega_{i,total} & =\omega \left\{T_{i}(a,\{{\overset{'}{M}}_{i1}(a),\ldots ,{\overset{'}{M}}_{i,p_{m}}(a)\})\right\}\\ & \;-\omega \left\{T_{i}(a^{\prime },\{{\overset{'}{M}}_{i1}(a^{\prime }),\ldots ,{\overset{'}{M}}_{i,p_{m}}(a^{\prime })\})\right\} \end{array}$$

where a and $a^{\prime }$ correspond to the two treatment arms. This conditional average total effect can be decomposed to a conditional direct effect defined as

(7) $$\begin{array}{ll} \Omega_{i,A\to T}(a) & =\omega \left\{T_{i}(a,\{{\overset{'}{M}}_{i1}(a),\ldots ,{\overset{'}{M}}_{i,p_{m}}(a)\})\right\}\\ & \;-\omega \left\{T_{i}(a^{\prime },\{{\overset{'}{M}}_{i1}(a),\ldots ,{\overset{'}{M}}_{i,p_{m}}(a)\})\right\} \end{array}$$

which is implemented along the direct causal pathway from the treatment to survival time, and a joint conditional indirect effect defined as

(8) $$\begin{array}{ll} \Omega_{i,A\to MT}(a^{\prime }) & =\omega \left\{T_{i}(a^{\prime },\{{\overset{'}{M}}_{i1}(a),\ldots ,{\overset{'}{M}}_{i,p_{m}}(a)\})\right\}\\ & \;-\omega \left\{T_{i}(a^{\prime },\{{\overset{'}{M}}_{i1}(a^{\prime }),\ldots ,{\overset{'}{M}}_{i,p_{m}}(a^{\prime })\})\right\} \end{array}$$

which is implemented by shifting the joint distribution of the potential mediators. Since the proposed model implicitly assumes no treatment–mediator interaction in the outcome regression equation, the two possible ways of decomposition, $\Omega_{i,\text{total}}=\Omega_{i,A\to T}(0)+\Omega_{i,A\to MT}(1)$ and $\Omega_{i,\text{total}}=\Omega_{i,A\to T}(1)+\Omega_{i,A\to MT}(0)$, should work equivalently up to sign.

Additionally, the conditional indirect effect through each mediator $M_{q}$ separately is defined as

(9) $$\begin{array}{ll} \Omega_{i,A\to M_{q}\to T}(a^{\prime }) & =\omega \left\{T_{i}(a^{\prime },{\overset{'}{M}}_{i1}(a^{\prime }),\ldots ,{\overset{'}{M}}_{i,q-1}(a^{\prime })\right.\\ & \;\left.{\overset{'}{M}}_{iq}(a),{\overset{'}{M}}_{i,q+1}(a^{\prime }),\ldots ,{\overset{'}{M}}_{i,p_{m}}(a^{\prime }))\right\}\\ & \;-\omega \left\{T_{i}(a^{\prime },{\overset{'}{M}}_{i1}(a^{\prime }),\ldots ,{\overset{'}{M}}_{i,p_{m}}(a^{\prime }))\right\} \end{array}$$

that is, by shifting the counterfactual marginal distribution of only the qth mediator from one treatment arm ($F_{M_{iq}(a^{\prime })|x_{i}}$) to the other ($F_{M_{iq}(a)|x_{i}}$), while keeping the remaining mediators unchanged. Existing studies employed the interventional (in)direct effect as a randomized interventional analogue of the natural (in)direct effect and focused on their population‐level interpretation through hypothetical interventions on A and M [31, 32]. Our definition above extends this approach to subpopulation or individual level, enabling the creation of randomized interventional analogues of the conditional average (in)direct effects. This extension allows us to interpret $\Omega_{i,A\to M_{q}\to T}(a^{\prime })$ as the effect that passing through the paths from the treatment to $M_{q}$ and then to the survival outcome directly, which is implemented through the hypothetical intervention that shifts its marginal distribution conditional on the covariate level of the ith subject. It is worth noting that such indirect effects through each mediator separately do not necessarily sum up to the joint indirect effect. Instead, the difference between the sum of the separate indirect effect and the joint indirect effect can be rewritten into two parts,

(10) $$\begin{array}{ll} & \omega \left\{T_{i}(a^{\prime },\{{\overset{'}{M}}_{i1}(a),\ldots ,{\overset{'}{M}}_{i,p_{m}}(a)\})\right\}\\ & \;-\omega \left\{T_{i}(a^{\prime },\{{\overset{'}{M}}_{i1}(a^{\prime }),\ldots ,{\overset{'}{M}}_{i,p_{m}}(a^{\prime })\})\right\}\\ & \;-\left[\omega \left\{T_{i}(a^{\prime },{\overset{'}{M}}_{i1}(a),\ldots ,{\overset{'}{M}}_{i,p_{m}}(a))\right\}\right.\\ & \;-\left.\omega \left\{T_{i}(a^{\prime },{\overset{'}{M}}_{i1}(a^{\prime }),\ldots ,{\overset{'}{M}}_{i,p_{m}}(a^{\prime }))\right\}\right] \end{array}$$

and

(11) $$\begin{array}{ll} & \omega \left\{T_{i}(a^{\prime },{\overset{'}{M}}_{i1}(a),\ldots ,{\overset{'}{M}}_{i,p_{m}}(a))\right\}\\ & \;-\omega \left\{T_{i}(a^{\prime },{\overset{'}{M}}_{i1}(a^{\prime }),\ldots ,{\overset{'}{M}}_{i,p_{m}}(a^{\prime }))\right\}\\ & \;-\overset{p_{m}}{\underset{q=1}{\sum }}\left[\omega \left\{T_{i}(a^{\prime },{\overset{'}{M}}_{i1}(a^{\prime }),\ldots ,{\overset{'}{M}}_{i,q-1}(a^{\prime })\right.\right.\\ & \;\left.{\overset{'}{M}}_{iq}(a),{\overset{'}{M}}_{i,q+1}(a^{\prime }),\ldots ,{\overset{'}{M}}_{i,p_{m}}(a^{\prime }))\right\}\\ & \;-\left.\omega \left\{T_{i}(a^{\prime },{\overset{'}{M}}_{i1}(a^{\prime }),\ldots ,{\overset{'}{M}}_{i,p_{m}}(a^{\prime }))\right\}\right] \end{array}$$

where the former is referred to as the indirect effect via the mediators' *mutual dependence* and the latter stands for a remainder effect [29, 30]. When the mediators are assumed to be causally unordered with no interactions in the outcome regression model, the indirect effect via the mediators' mutual dependence and the remainder effect become zero. In this scenario, the interventional joint indirect effect can be “decomposed” into the separate indirect effects via each mediator. Besides, for the simple single‐mediator case with $p_{m}=1$, the joint indirect effect just coincides with the indirect effect through M, that is,

(12) $$\Omega_{i,A\to M\to T}(a^{\prime })=\omega \left\{T_{i}(a^{\prime },M_{i}(a))\right\}-\omega \left\{T_{i}(a^{\prime },M_{i}(a^{\prime }))\right\}$$

with the additional effect in Equations (10) and (11) reducing to zero.

To identify the targeted functions ω{·} from the proposed model, we invoke a series of sequential ignorability assumptions:

Assumption 1 $T_{i}(a,m_{1},\ldots ,m_{p_{m}})╨A_{i}\;|\;x_{i}=x$, that is, there is no unmeasured confounders for the  causal pathway from the treatment to the time‐to‐event outcome conditional on $x_{i}$.

Assumption 2 $T_{i}(a,m_{1},\ldots ,m_{p_{m}})╨M_{i}={\left(M_{i1},\ldots ,M_{i,p_{m}}\right)}^{T}\;|\;A_{i},\;x_{i}=x$, that is, there is no unmeasured confounders for the causal pathway from the mediator(s) to the time‐to‐event outcome conditional on $A_{i}$ and $x_{i}$.

Assumption 3 $M_{i}(a)={\left(M_{i1}(a),\ldots ,M_{i,p_{m}}(a)\right)}^{T}╨A_{i}\;|\;x_{i}=x$, that is, there is no unmeasured confounders for the causal pathway from the treatment to the mediator(s) conditional on $x_{i}$.

“A ╨ B | C” in the above assumptions denotes independence between A and B conditional on C, and a∈{0,1}. Under these assumptions, $\omega \left\{T_{i}(a,{\overset{'}{M}}_{i1}(a_{1}),\ldots ,{\overset{'}{M}}_{i,p_{m}}(a_{p_{m}}))\right\}$ can be identified nonparametrically as

(13) $$\begin{array}{ll} & \omega \left\{T_{i}(a,{\overset{´}{M}}_{i1}(a_{1}),\ldots ,{\overset{´}{M}}_{i,p_{m}}(a_{p_{m}}))\right\}\\ & \;=\int \ldots \int \omega \left\{T_{i}|A_{i}=a,M_{i1}=m_{1},\ldots ,M_{i,p_{m}}\right.=\left.m_{p_{m}},x_{i}=x\right\}\\ & \;\;\;\;\;\;dF_{M_{i1}|A_{i}=a_{1},x_{i}=x}\ldots dF_{M_{i,p_{m}}|A_{i}=a_{p_{m}},x_{i}=x} \end{array}$$

where $F_{M_{iq}|A_{i},x_{i}}$, $q=1,\ldots ,p_{m}$, denotes the marginal distribution function of $M_{q}$ conditional on treatment level A and covariate x. The integration over $F_{M_{iq}|A_{i},x_{i}}$ can be approximated through Monte Carlo integration, where the integrand is calculated and averaged over random realizations of $M_{iq}$ in the treatment arm $a_{q}$ simulated from the corresponding posterior samples. Similarly, $\omega \left\{T_{i}(a,\{{\overset{'}{M}}_{i1}(a^{\prime }),\ldots ,{\overset{'}{M}}_{i,p_{m}}(a^{\prime })\})\right\}$ can be identified as

(14) $$\begin{array}{ll} & \omega \left\{T_{i}(a,\{{\overset{´}{M}}_{i1}(a^{\prime }),\ldots ,{\overset{´}{M}}_{i,p_{m}}(a^{\prime })\})\right\}\\ & \;=\int \omega \left\{T_{i}|A_{i}=a,M_{i1}=m_{1},\ldots ,M_{i,p_{m}}=\left.m_{p_{m}},x_{i}=x\right\}\right.\\ & \;\;\;\;\;\;dF_{M_{i}|A_{i}=a^{\prime },x_{i}=x} \end{array}$$

where $F_{M_{i}|A_{i},x_{i}}$ denotes the joint distribution function of $M_{i}$ conditional on treatment level A and covariate x. The group‐level or population‐level average mediation effect, which is at the heart of conventional mediation analysis, can be identified through

(15) $$\begin{array}{ll} & \bar{\omega }\left\{T_{i}(a,{\overset{´}{M}}_{i1}(a_{1}),\ldots ,{\overset{´}{M}}_{i,p_{m}}(a_{p_{m}}))\right\}\\ & \;=E\left[\bar{\omega }\left\{T_{i}(a,{\overset{´}{M}}_{i1}(a_{1}),\ldots ,{\overset{´}{M}}_{i,p_{m}}(a_{p_{m}}))\right\}\right]\\ & \;=\iint \cdots \int \omega \left\{T_{i}|A_{i}=a,M_{i1}=m_{1},\ldots ,M_{i,p_{m}}=\left.m_{p_{m}},x_{i}=x\right\}\right.\\ & \;\;\;\;\;\;dF_{M_{i1}|A_{i}=a_{1},x_{i}=x}\cdots dF_{M_{i,p_{m}}|A_{i}=a_{p_{m}},x_{i}=x}dF_{x_{i}} \end{array}$$

and

(16) $$\begin{array}{ll} & \bar{\omega }\left\{T_{i}(a,\{{\overset{´}{M}}_{i1}(a^{\prime }),\ldots ,{\overset{´}{M}}_{i,p_{m}}(a^{\prime })\})\right\}\\ & \;=E\left[\omega \left\{T_{i}(a,\{{\overset{´}{M}}_{i1}(a^{\prime }),\ldots ,{\overset{´}{M}}_{i,p_{m}}(a^{\prime })\})\right\}\right]\\ & \;=\iint \omega \left\{T_{i}|A_{i}=a,M_{i1}=m_{1},\ldots ,M_{i,p_{m}}=\left.m_{p_{m}},x_{i}=x\right\}\right.\\ & \;\;\;\;dF_{M_{i}|A_{i}=a^{\prime },x_{i}=x}dF_{x_{i}} \end{array}$$

where $F_{x_{i}}$ denotes the distribution function of covariates x. The integration is often approximated by averaging the function of the potential survival time identified in (13) and (14) over the empirical distribution of x to avoid separate modelling of the covariate distribution. Derivation of Equations ((13), (14), (15), (16)) under the assumptions outlined above is provided in Appendix A of the Supporting Information.

The proposed model links the causal pathways from the treatment and the mediators to the time‐to‐event outcome through a PH model. Consequently, the logarithm of hazards serves as a natural choice for the targeted function ω{·}. Other options are also viable, including survival probability [33], transformation of survival times [2, 6], and restricted mean survival time [4, 5], each offering a different perspective on the survival outcome. This study focuses on the hazard function and the survival probability as illustrative examples of the targeted function ω{·} to demonstrate the estimation of the ICPSEs.

### 3.1 ICPSEs on Logarithm of Hazards

With $\omega^{\lambda }\left\{T_{i}(a,\{{\overset{'}{M}}_{i1}(a^{\prime }),\ldots ,{\overset{'}{M}}_{i,p_{m}}(a^{\prime })\})\right\}=\log\lambda (T_{i}(a,\{{\overset{'}{M}}_{i1}(a^{\prime }),\ldots ,{\overset{'}{M}}_{i,p_{m}}(a^{\prime })\}))$, we define the ICPSEs on the logarithm of hazards as follows:

(17) $$\begin{array}{ll} \Omega_{i,A\to T}^{\lambda }(1) & =\log\lambda \left(T_{i}(1,\{{\overset{'}{M}}_{i1}(1),\ldots ,{\overset{'}{M}}_{i,p_{m}}(1)\})\right)\\ & \;-\log\lambda \left(T_{i}(0,\{{\overset{'}{M}}_{i1}(1),\ldots ,{\overset{'}{M}}_{i,p_{m}}(1)\})\right)\\ \Omega_{i,A\to MT}^{\lambda }(0) & =\log\lambda \left(T_{i}(0,\{{\overset{'}{M}}_{i1}(1),\ldots ,{\overset{'}{M}}_{i,p_{m}}(1)\})\right)\\ & \;-\log\lambda \left(T_{i}(0,\{{\overset{'}{M}}_{i1}(0),\ldots ,{\overset{'}{M}}_{i,p_{m}}(0)\})\right) \end{array}$$

whereas the conditional average total effect on the logarithm of hazards adds up to

(18) $$\begin{array}{ll} \Omega_{i,\text{total}}^{\lambda } & =\Omega_{i,A\to T}^{\lambda }(1)+\Omega_{i,A\to MT}^{\lambda }(0)\\ & =\log\lambda \left(T_{i}(1,\{{\overset{'}{M}}_{i1}(1),\ldots ,{\overset{'}{M}}_{i,p_{m}}(1)\})\right)\\ & \;-\log\lambda \left(T_{i}(0,\{{\overset{'}{M}}_{i1}(0),\ldots ,{\overset{'}{M}}_{i,p_{m}}(0)\})\right) \end{array}$$

Similarly, with $\omega^{\lambda }\left\{T_{i}(a,{\overset{'}{M}}_{i1}(a_{1}),\ldots ,{\overset{'}{M}}_{i,p_{m}}(a_{p_{m}}))\right\}=\log\lambda (T_{i}(a,{\overset{'}{M}}_{i1}(a_{1}),\ldots ,{\overset{'}{M}}_{i,p_{m}}(a_{p_{m}})))$, the conditional indirect effect through each mediator separately is given by

(19) $$\begin{array}{ll} & \Omega_{i,A\to M_{q}\to T}^{\lambda }(0)\\ & \;=\log\lambda \left(T_{i}(0,{\overset{'}{M}}_{i1}(0),\ldots ,{\overset{'}{M}}_{i,q-1}(0),\right.\\ & \;\left.{\overset{'}{M}}_{iq}(1),{\overset{'}{M}}_{i,q+1}(0),\ldots ,{\overset{'}{M}}_{i,p_{m}}(0))\right)\\ & \;-\log\lambda \left(T_{i}(0,{\overset{'}{M}}_{i1}(0),\ldots ,{\overset{'}{M}}_{i,p_{m}}(0))\right),\\ & \;\;q=1,\ldots ,p_{m} \end{array}$$

The counterfactual logarithm of hazards, $\log\lambda \left(T_{i}(a,{\overset{'}{M}}_{i1}(a_{1}),\ldots ,{\overset{'}{M}}_{i,p_{m}}(a_{p_{m}}))\right)$ and $\log\lambda (T_{i}(a,\{{\overset{'}{M}}_{i1}(a^{\prime }),\ldots ,{\overset{'}{M}}_{i,p_{m}}(a^{\prime })\}))$, are identified from the proposed model as given in (13) and (14).

### 3.2 ICPSEs on Survival Probability

Based on the PH model, the survival function at a a given time $t^{*}$ can be expressed as

(20) $$\begin{array}{ll} S(t^{*}|x,M,A) & =\exp\{-\int_{0}^{t^{*}}\lambda (u|x,M,A)du\}\\ & =\exp\{-\int_{0}^{t^{*}}\lambda_{0}(u)\exp\left(\upsilon (x,M)+\tau (x)A\right)du\} \end{array}$$

With $\omega^{S}\left\{T_{i}(a,\{{\overset{'}{M}}_{i1}(a^{\prime }),\ldots ,{\overset{'}{M}}_{i,p_{m}}(a^{\prime })\})\right\}=S(T_{i}(a,\{{\overset{'}{M}}_{i1}(a^{\prime }),\ldots ,{\overset{'}{M}}_{i,p_{m}}(a^{\prime })\}))$, and $\omega^{S}\{T_{i}(a,{\overset{'}{M}}_{i1}(a_{1}),\ldots ,{\overset{'}{M}}_{i,p_{m}}(a_{p_{m}}))\}=S(T_{i}(a,{\overset{'}{M}}_{i1}(a_{1}),\ldots ,{\overset{'}{M}}_{i,p_{m}}(a_{p_{m}})))$, the ICPSEs and total effect on the probability of surviving over $t^{*}$ are defined as

(21) $$\begin{array}{ll} \Omega_{i,A\to T}^{S}(1) & =S(T_{i}(1,\{{\overset{'}{M}}_{i1}(1),\ldots ,{\overset{'}{M}}_{i,p_{m}}(1)\}))\\ & \;-S(T_{i}(0,\{{\overset{'}{M}}_{i1}(1),\ldots ,{\overset{'}{M}}_{i,p_{m}}(1)\}))\\ \Omega_{i,A\to MT}^{S}(0) & =S(T_{i}(0,\{{\overset{'}{M}}_{i1}(1),\ldots ,{\overset{'}{M}}_{i,p_{m}}(1)\}))\\ & \;-S(T_{i}(0,\{{\overset{'}{M}}_{i1}(0),\ldots ,{\overset{'}{M}}_{i,p_{m}}(0)\}))\\ \Omega_{i,\text{total}}^{S} & =S(T_{i}(1,\{{\overset{'}{M}}_{i1}(1),\ldots ,{\overset{'}{M}}_{i,p_{m}}(1)\}))\\ & \;-S(T_{i}(0,\{{\overset{'}{M}}_{i1}(0),\ldots ,{\overset{'}{M}}_{i,p_{m}}(0)\}))\\ \Omega_{A\to M_{q}\to T}^{S}(0) & =S\left(T_{i}(0,{\overset{'}{M}}_{i1}(0),\ldots ,{\overset{'}{M}}_{i,q-1}(0),\right.\\ & \;\left.{\overset{'}{M}}_{iq}(1),{\overset{'}{M}}_{i,q+1}(0),\ldots ,{\overset{'}{M}}_{i,p_{m}}(0))\right)\\ & \;-S\left(T_{i}(0,{\overset{'}{M}}_{i1}(0),\ldots ,{\overset{'}{M}}_{i,p_{m}}(0))\right),\\ & \;q=1,\ldots ,p_{m} \end{array}$$

## 4 Bayesian Analysis

### 4.1 Prior Specification

Let $Y={\left(Y_{1},Y_{2},\ldots ,Y_{n}\right)}^{T}$, $M={\left(M_{1},M_{2},\ldots ,M_{n}\right)}^{T}$, $A={\left(A_{1},A_{2},\ldots ,A_{n}\right)}^{T}$, $X={\left(x_{1}^{T},x_{2}^{T},\ldots ,x_{n}^{T}\right)}^{T}$, and $\delta ={\left(\delta_{1},\ldots ,\delta_{n}\right)}^{T}$. Let 𝒟={Y,X,A,δ,M} denote the observed data. The complete data likelihood of the proposed model is expressed as

(22) $$\begin{array}{ll} p(𝒟|\cdot ) & =\prod_{i=1}^{n}\exp[-\Lambda_{0}(Y_{i})\exp\{\upsilon (x_{i},M_{i})+\tau (x_{i})A_{i}\}]\\ & \;\times {\left[\lambda_{0}(Y_{i})\exp\{\upsilon (x_{i},M_{i})+\tau (x_{i})A_{i}\}\right]}^{\delta_{i}}\\ & \;\times {\left(2\pi \right)}^{-p_{m}/2}\det{\left(\Sigma \right)}^{-1/2}\exp\left\{-\frac{1}{2}\left(M_{i}-\upsilon_{M}(x_{i})\right.\right.\\ & \;\;\;\;\;\;-\left.{\left.\tau_{M}(x_{i})A_{i}\right)}^{T}\Sigma^{-1}\left(M_{i}-\upsilon_{M}(x_{i})-\tau_{M}(x_{i})A_{i}\right)\right\} \end{array}$$

where $\Lambda_{0}(t)=\int_{0}^{t}\lambda_{0}(u)du$ is the cumulative baseline hazard function. We adopt Bayesian P‐splines [34] to achieve flexible estimation of the baseline hazard and the ICPSEs. The basic idea is to approximate $\lambda_{0}(t)$ through a set of B‐spline basis functions, i.e.,

(23) $$\lambda_{0}(t)=\exp\left(\overset{L}{\underset{l=1}{\sum }}\gamma_{l}B_{l}(t)\right)=\exp\left(\gamma^{T}B(t)\right)$$

where L is the number of B‐spline segments determined by a prespecified number of knots on $\left[0,\max_{i}Y_{i}\right]$, $B(t)={\left(B_{1}(t),\ldots ,B_{L}(t)\right)}^{T}$ is the corresponding set of cubic B‐spline basis functions, and $\gamma ={\left(\gamma_{1},\ldots ,\gamma_{L}\right)}^{T}$ is the vector of unknown coefficients. Following the common practice in survival analysis [4, 35], we set 10 equidistant knots on $\left[0,\max_{i}Y_{i}\right]$ and applied a roughness penalty on coefficients of the B‐splines to counterbalance its flexibility and avoid overfitting. The penalty is imposed on the (higher‐order) finite differences of adjacent B‐splines coefficients. We considered a second‐order difference penalty of the form: $\rho \sum_{l}{\left(\Delta^{2}\gamma_{l}\right)}^{2}=\rho \gamma^{T}D^{T}D\gamma$, where ρ is the penalty parameter that controls smoothness of the fit and $\Delta^{2}$ is the second‐order difference operator with a matrix representation

(24) $$D=\left(\begin{array}{cccccc} 1 & -2 & 1 & & & \\ & 1 & -2 & 1 & & \\ & & \ddots & \ddots & \ddots & \\ & & & 1 & -2 & 1 \end{array}\right)$$

Accounting for this penalty, the prior distribution for the B‐splines coefficients was specified as $p(\gamma |\rho )\propto \exp\left(-\frac{2}{\rho }\gamma^{T}P\gamma \right)$, that is, a multivariate Gaussian distribution with a precision matrix $P=D^{T}D+10^{-3}I$. We assigned a gamma hyperprior for the penalty parameter, that is, $\rho ∼\text{Gamma}(a_{0},b_{0})$, where $a_{0}$ and $b_{0}$ are prespecified hyperparameters. A common choice is $a_{0g}=1$ and $b_{0g}=0.001$, which produces a dispersed prior.

Assuming prior independence among the individual trees and the residual covariance matrix Σ, the prior distribution for the remaining parameters within the tree ensembles can be formulated as follows:

(25) $$\begin{array}{ll} & p\left({\left\{{𝒯}_{j},{ℳ}_{j}\right\}}_{j=1}^{J},{\left\{{\tilde{𝒯}}_{h},{\tilde{ℳ}}_{h}^{\left(M\right)},{\tilde{ℳ}}_{h}\right\}}_{h=1}^{H},{\left\{{\overset{˘}{𝒯}}_{k},{\overset{˘}{ℳ}}_{k}\right\}}_{k=1}^{K},\Sigma \right)\\ & \;=\overset{J}{\underset{j=1}{\prod }}p({𝒯}_{j},{ℳ}_{j})\overset{H}{\underset{h=1}{\prod }}p({\tilde{𝒯}}_{h},{\tilde{ℳ}}_{h}^{\left(M\right)},{\tilde{ℳ}}_{h})\overset{K}{\underset{k=1}{\prod }}p({\overset{˘}{𝒯}}_{k},{\overset{˘}{ℳ}}_{k})p(\Sigma )\\ & \;=\overset{J}{\underset{j=1}{\prod }}\overset{b_{j}}{\underset{l=1}{\prod }}p(\mu_{jl}|{𝒯}_{j})p({𝒯}_{j})\overset{H}{\underset{h=1}{\prod }}\overset{b_{h}}{\underset{l=1}{\prod }}p({\tilde{\mu }}_{hl}^{\left(M\right)}|{\tilde{𝒯}}_{h})\\ & \;p({\tilde{\mu }}_{hl}|{\tilde{𝒯}}_{h})p({\tilde{𝒯}}_{h})\overset{K}{\underset{k=1}{\prod }}\overset{b_{k}}{\underset{l=1}{\prod }}p({\overset{˘}{\mu }}_{kl}|{\overset{˘}{𝒯}}_{k})p({\overset{˘}{𝒯}}_{k})p(\Sigma ) \end{array}$$

Among the hyperparameters $\{\alpha ,\beta ,\tilde{\alpha },\tilde{\beta },\overset{˘}{\alpha },\overset{˘}{\beta },c_{1},c_{2},k_{1},k_{2},\overset{˘}{\zeta },$ $\overset{˘}{\eta },\tilde{\zeta },\tilde{\eta },\varkappa ,\alpha_{D}\}$, the αs and βs restrict the depth of each individual binary tree through $p({𝒯}_{j})$, $p({\tilde{𝒯}}_{h})$, and $p({\overset{˘}{𝒯}}_{k})$, ϰ and $\alpha_{D}$ controls the variable selection process through the splitting rules, while the rest confine the prior probability for $\upsilon_{M}(x)$, $\tau_{M}(x)$, τ(x), and υ(x) through $p(\mu_{jl}|{𝒯}_{j})$, $p({\tilde{\mu }}_{hl}^{\left(M\right)}|{\tilde{𝒯}}_{h})$, $p({\tilde{\mu }}_{hl}|{\tilde{𝒯}}_{h})$, and $p({\overset{˘}{\mu }}_{kl}|{\overset{˘}{𝒯}}_{k})$, respectively. For the prognostic functions $\upsilon_{M}(x)$ and υ(x), we set $\alpha =\overset{˘}{\alpha }=0.95$ and $\beta =\overset{˘}{\beta }=2$ such that shallow trees with two or three leaf nodes are allowed with higher prior probability. For $\tau_{M}(x)$ and τ(x) that capture the direct effect of the treatment through shared tree topology, we set $\tilde{\alpha }=0.95$ and $\tilde{\beta }=3$ to grow even shallower individual trees to avoid false discovery of the effect modifiers or heterogeneity in the ICPSEs. Different combinations of commonly used BART priors are also explored in a sensitivity analysis to evaluate the robustness of the estimated causal effects. For the sparsity‐inducing Gibbs‐type prior on the splitting rules, we follow the default setup of Linero and Du [21] to set $\varkappa =\alpha_{D}=1$.

For the mediator regression equations, we assign a conjugate inverse‐Wishart prior to the residual covariance matrix, $\Sigma ∼IW(\Psi^{-1},\nu )$, where Ψ is a $p_{m}\times p_{m}$ diagonal matrix. To set the diagonal elements of Ψ, we first get a rough estimate of the residual variance for each mediator, denoted as ${\hat{\sigma }}_{rq}^{2}$, by performing an ordinary least squared (OLS) regression of $M_{q}$ on A and x. ${\hat{\sigma }}_{rq}^{2}$ is expected to overestimate the qth diagonal element of Σ, that is, $\sigma_{q}^{2}$, given that no higher‐order terms, such as interactions, is considered in the OLS regression. Therefore, we assign an inverse‐chi‐squared prior, $\sigma_{q}^{2}∼\kappa_{q}\nu /\chi_{\nu }^{2}$, where $\kappa_{q}$ is chosen to ensure that the prior probability of $\sigma_{q}^{2}$ being greater than the rough estimate ${\hat{\sigma }}_{rq}^{2}$ is controlled at around 1−ϱ. ϱ∈(0,1) is a prespecified constant that adjusts the comparative scale of $\sigma_{q}^{2}$ based on ${\hat{\sigma }}_{rq}^{2}$, and a common choice is 0.95. The degree of freedom hyperparameter of the inverse‐chi‐squared prior is set as ν=3 as suggested by Chipman, George, and McCulloch [19]. The marginal inverse‐chi‐squared priors then leads to $\Psi =\text{diag}(\kappa_{1},\ldots ,\kappa_{p_{m}})$. For the leaf node hyperparameters ${\left\{{ℳ}_{j}\right\}}_{j=1}^{J}$ and ${\left\{{\tilde{ℳ}}_{h}^{\left(M\right)}\right\}}_{h=1}^{H}$, we adopt the common practice to set $c_{1}=c_{2}=4$ and the qth diagonal element of $\Sigma_{0}$ and ${\tilde{\Sigma }}_{0}$ as the sample SD of $M_{q}$, denoted by ${\hat{\sigma }}_{0q}^{2}$. This results in a normal prior, $N\left(0,\frac{4{\hat{\sigma }}_{0q}^{2}}{k_{1}^{2}}\right)$, for the qth element of the prognostic function $\upsilon_{M}(x)$, such that the interval $\left(-\frac{4{\hat{\sigma }}_{0q}}{k_{1}},\frac{4{\hat{\sigma }}_{0q}}{k_{1}}\right)$ covers 95% of its prior probability. Similarly, a normal prior $N\left(0,\frac{4{\hat{\sigma }}_{0q}^{2}}{k_{2}^{2}}\right)$ is assigned to the qth element of $\tau_{M}(x)$, which is the conditional average effect of A on $M_{q}$ characterized by the sum of H leaf node parameters ${\tilde{\mu }}_{hl,q}^{\left(M\right)}$s. By choosing $k_{1}=2$ and $k_{2}=4$, the normal priors control the (marginal) prior distribution of each expected mediator under treatment within a range of $\pm 3{\hat{\sigma }}_{0q}$ with a probability of around 99.6%.

For the leaf node parameters ${\left\{{\overset{˘}{ℳ}}_{k}\right\}}_{k=1}^{K}$ in the PH model, we assign conjugate log‐gamma priors, ${\overset{˘}{\mu }}_{kl}∼logGam(\overset{˘}{\zeta },\overset{˘}{\eta })$. Following Linero et al. [36], we introduce regularization to homogeneity on the prognostic functions by setting $\log\overset{˘}{\eta }=\psi (\overset{˘}{\zeta })$, where $\psi (\overset{˘}{\zeta })$ is the digamma function, such that ${\overset{˘}{\mu }}_{kl}$ is controlled with a prior mean of 0 and a prior variance of $\sigma_{\overset{˘}{\mu }}^{2}=\psi^{\prime }(\overset{˘}{\zeta })$. We follow the suggestions therein to set $\sigma_{\overset{˘}{\mu }}∼\text{Half‐Cauchy}(0,1.5/\sqrt{K}))$ and further adopt a simple approximation of $\overset{˘}{\zeta }=\sigma_{\overset{˘}{\mu }}^{-2}+0.5$ and $\overset{˘}{\eta }=\sigma_{\overset{˘}{\mu }}^{-2}$, as proposed by Murray [37], to ease the numerical calculation of the digamma and trigamma functions. The same set of procedures is also implemented for ${\tilde{\mu }}_{hl}∼logGam(\tilde{\zeta },\tilde{\eta })$, but with stronger regularization to homogeneity introduced by $\sigma_{\tilde{\mu }}∼\text{Half‐Cauchy}(0,0.5/\sqrt{H})$.

We set J=K=200 for the prognostic functions in both the mediator regression model and the PH model, which is a commonly used default for BART‐based approaches. For τ(x) and $\tau_{M}(x)$, which characterize the direct pathways from the treatment, we consider H=100 to accommodate varying overlapped structure in the effect modifiers. Different choices for the above set of hyperparameters are studied in the simulation studies to check stability of the proposed method under different prior regularizations on heterogeneity.

### 4.2 Posterior Inference

Within the Bayesian framework, the estimation of the ICPSEs is facilitated through posterior sampling from the full conditional distributions of the binary trees, which are derived from the complete‐data likelihood in Equation (22) and the prior distributions outlined above. We employ a Bayesian Backfitting MCMC algorithm in tandem with the Gibbs sampler to implement efficient sampling. This integrated procedure allows for sequential updates of the individual binary trees and nuisance parameters, as outlined in Algorithm 1. A detailed derivation of the full conditional distributions is provided in Appendix B of the Supporting Information. Additionally, a multivariate extension of the comonotonic sampling strategy [27] is adopted to enhance the numerical implementation of the mediation formula. The details of this extended sampling strategy are presented in Algorithms S1 of Appendix A in Supporting Information.

ALGORITHM 1 **The hybrid MCMC algorithm with Bayesian backfitting and Gibbs sampler.**

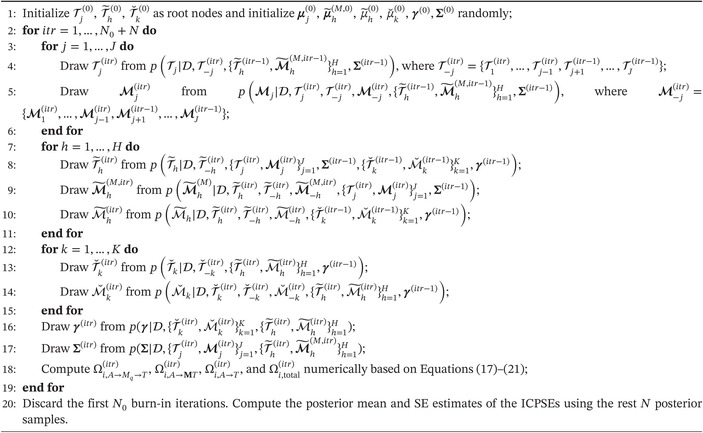

Leveraging the posterior samples of the ICPSEs, we can assess the evidence of heterogeneity in each causal pathway by evaluating the posterior probability of differential treatment effects, as proposed by Henderson et al. [9]. Specifically, let $D_{i,A\to T}=Pr\left(\Omega_{i,A\to T}\geq {\bar{\Omega }}_{A\to T}|𝒟\right)$, where ${\bar{\Omega }}_{A\to T}=\frac{1}{n}\sum_{i=1}^{n}\Omega_{i,A\to T}$ denotes the sample average direct effect, and let $D_{i,A\to T}^{*}=\max\{1-2D_{i,A\to T},2D_{i,A\to T}-1\}$. Across the MCMC iterations, an individualized direct effect that deviates from the sample average level becomes evident when the corresponding value of $D_{i,A\to T}^{*}$ approaches 1, or when $D_{i,A\to T}$ is close to either 0 or 1. Similar indices, $D_{i,A\to M_{q}\to T}\left(D_{i,A\to M_{q}\to T}^{*}\right)$, $D_{i,A\to MT}\left(D_{i,A\to MT}^{*}\right)$, and $D_{i,\text{total}}\left(D_{i,\text{total}}^{*}\right)$, can be defined for the separate and joint indirect effects, as well as the total effect. Following the recommendations of Henderson et al. [9] and Chen et al. [38], we consider $D_{i,\cdot }^{*}>0.9$ (i.e., $D_{i,\cdot }>0.95$ or $D_{i,\cdot }<0.05$) as strong evidence of individual‐specific differential PSEs, $D_{i,\cdot }^{*}>0.8$ (i.e., $D_{i,\cdot }>0.9$ or $D_{i,\cdot }<0.1$) as moderate evidence, and $D_{i,\cdot }^{*}>0.7$ (i.e., $D_{i,\cdot }>0.85$ or $D_{i,\cdot }<0.15$) as mild evidence. The potential sources of heterogeneity can be evaluated by examining the posterior splitting proportions of the predictors in $\tau_{M}(x_{i})$, $\tau (x_{i})$, and $\upsilon (x_{i},M_{i})$ across the iterations, which are readily available from the collected posterior draws of the corresponding binary trees. To further elucidate the specific manner in which the top‐selected effect modifiers induce heterogeneity on each causal pathway, we can leverage group‐average ICPSEs and the partial effect of certain covariates of interest, denoted by $x_{s}$. The partial effect of $x_{s}$ can be estimated by averaging the ICPSEs over the samples in which $x_{s}$ is fixed at a set of reasonable values.

## 5 Simulation Study

In this section, we assessed the finite sample performance of the proposed method in estimating the ICPSEs under a multiple‐mediator case with $p_{m}=2$. We generated p=25 correlated covariates from a multivariate Gaussian distribution, x∼𝒩(0,Σ), with the entries of the covariance matrix Σ being $\Sigma (j,k)=0.3^{\left|j-k\right|}+0.1I(j\neq k)$. The prognostic functions and true direct effects on the mediators and hazard of the survival outcome were set as

$$\begin{array}{ll} & \text{setup (i):}\;\upsilon_{M}(x_{i})=\left(\begin{array}{c} \upsilon_{1}(x_{i})\\ \upsilon_{2}(x_{i}) \end{array}\right)\\ & \;\;\;\;\;\;\;\;\;\;\;\;\;\;\;\;\;\;\;\;\;\;\;\;\;\;\;=\left(\begin{array}{c} 0.15{\left(x_{i4}-2\right)}^{2}+|x_{i3}-1|\\ -\;0.5+0.5|x_{i3}-1|+0.5|x_{i4}-1|+\cos(\pi x_{i9}) \end{array}\right)\\ & \;\;\;\;\;\;\;\;\;\;\;\;\;\;\;\;\;\upsilon (x_{i},M_{i})=-0.2|x_{i3}|+0.5x_{i,12}+M_{i1}\\ & \;+(1+0.5I(x_{i6}>0.5))M_{i2}\\ & \;\;\;\;\;\;\;\;\;\;\;\;\;\;\;\;\;\tau_{M}(x_{i})=\left(\begin{array}{c} -\;1+0.5x_{i2}-0.5|x_{i7}|\\ -\;1+0.5x_{i7}-0.25\log(|x_{i5}|) \end{array}\right)\\ & \;\;\;\;\;\;\;\;\;\;\;\;\;\;\;\;\;\tau (x_{i})=-0.5+|x_{i6}|+\sqrt{\left|(x_{i6}-2)x_{i2}\right|} \end{array}$$

The true propensity score for each subject was generated by

$$\begin{array}{ll} & \pi (x_{i})\overset{\Delta }{=}Pr(A_{i}=1|x_{i})\\ & \;=0.8\Phi \left(\frac{\begin{array}{c} 0.2\times (-1+|x_{i3}-1|+|x_{i4}-1|) \end{array}}{s_{\pi }}-0.1x_{i1}\right)\\ & \;+0.05+0.1\xi_{i} \end{array}$$

where $s_{\pi }$ is the sample SD of the function on the numerator and $\xi_{i}\overset{i.i.d}{∼}\text{Uniform}(0,1)$ is a noise component. We simulated the event times with a true baseline hazard of $\lambda_{0}(t)=1$. The mediators were generated with a true residual variance–covariance matrix of $\text{Cov}(\epsilon_{i})=\left[\begin{array}{ll} 1 & 0.3\\ 0.3 & 1 \end{array}\right]$. We considered two sample sizes, n=1000 and n=2000. For each scenario, 100 replications were conducted. Prior inputs were specified as described in Section 4.1, and the unknown baseline hazard function $\lambda_{0}(t)$ was approximated using cubic B‐splines with 10 equidistant knots. Following common practice, we included the estimated propensity scores $\hat{\pi }(x_{i})$ as an additional covariate/predictor for the prognostic functions $\upsilon_{M}(x_{i})$ and $\upsilon (x_{i},M_{i})$ to alleviate regularization‐induced confounding.

**FIGURE 1:**
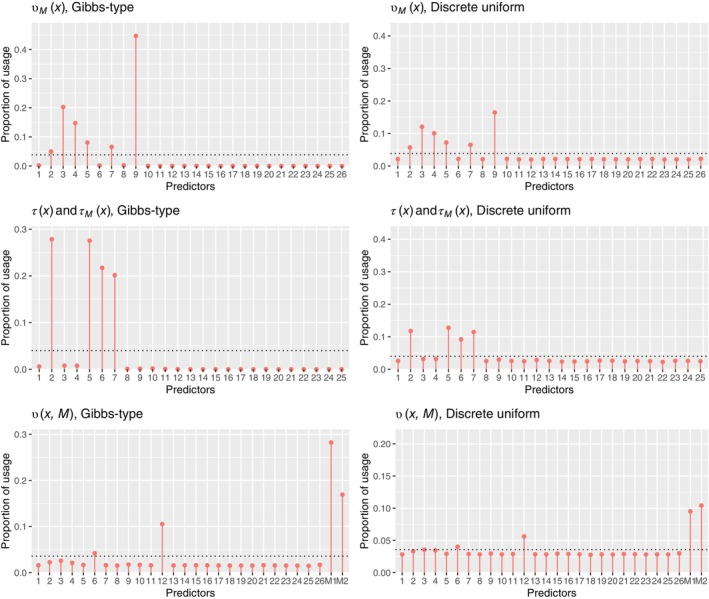
Posterior splitting proportions in the tree ensembles for each covariate under the Gibbs‐type prior (left) and the default discrete uniform prior (right) in setup (i) with n=1000. The horizontal dotted lines stand for the discrete uniform splitting probabilities.

To compute the Bayesian estimates of the ICPSEs, we ran the MCMC algorithm with 2000 iterations after a burn‐in stage of 1000 iterations and conducted Monte Carlo implementation of the mediation formula with $K_{M}=1$ through comonotonic sampling within each iteration. Figure S1 depicts the distribution of the true and estimated ICPSEs on each scale based on one randomly selected replication. The distribution of the individualized ICPSEs across the causal pathways are covered by the proposed method with satisfying accuracy and the sample average interventional effects are estimated precisely. We evaluated the performance of the proposed methodology using both average‐level and individual‐level criteria, which have been adopted in existing works on heterogeneous mediation analysis [27, 39]. At average level, we computed the bias, relative bias, and root mean squared error (RMSE) for the sample average interventional PSEs regarding each causal pathway. At individual level, we reported the squared root of the precision in estimating heterogeneous effects (PEHE) [40]. For example, for the direct path, $\sqrt{\text{PEHE}}$ was calculated as $\sqrt{\frac{1}{n}\sum_{i=1}^{n}{\left(\Omega_{i,A\to T}-{\hat{\Omega }}_{i,A\to T}\right)}^{2}}$. Table 1 presents the average bias, relative bias, and RMSE for the estimated sample average interventional PSEs, as well as the average $\sqrt{\text{PEHE}}$ based on the 100 replications. The default discrete uniform prior of BART‐based models were also performed for comparison. The proposed method with Gibbs‐type prior produced better estimates of the ICPSEs, as evidenced by the substantially smaller bias, RMSE, and $\sqrt{\text{PEHE}}$ values. As expected, an increasing sample size led to improved estimation results for both methods, but the proposed method consistently outperformed the default one across different sample sizes. Figure 1 shows the average posterior splitting proportions of the predictors in each tree ensemble based on the replications. The true confounders and effect modifiers were selected with high probabilities by the proposed method, while irrelevant variables were almost excluded with near‐zero probabilities.

**TABLE 1:** Average bias, relative bias, root mean squared error (RMSE) for the sample average interventional path‐specific effects (PSEs), and $\sqrt{\text{PEHE}}$ for the interventional conditional path‐specific effects (ICPSEs) on the scale of logarithm of hazards and survival probability at the mean observed event time under setup (i) with two mediators.

		n=1000				n=2000			
		Gibbs type		Discrete uniform		Gibbs type		Discrete uniform	
PSE	Criterion	Logh	Survp	Logh	Survp	Logh	Survp	Logh	Survp
DE	Bias	0.085	−0.006	0.215	−0.022	0.056	−0.005	0.141	−0.013
	RBias	0.115	0.086	0.254	0.264	0.078	0.061	0.192	0.150
	RMSE	0.117	0.012	0.267	0.033	0.078	0.009	0.153	0.020
	$\sqrt{\text{PEHE}}$	0.387	0.061	0.513	0.080	0.305	0.049	0.392	0.063
IE 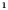	Bias	−0.090	0.013	−0.257	0.036	−0.070	0.009	−0.163	0.020
	RBias	0.072	0.094	0.197	0.237	0.057	0.068	0.130	0.145
	RMSE	0.119	0.017	0.291	0.039	0.091	0.012	0.187	0.024
	$\sqrt{\text{PEHE}}$	0.405	0.058	0.593	0.076	0.308	0.046	0.436	0.059
IE 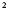	Bias	−0.037	0.009	−0.047	0.012	−0.027	0.005	−0.045	0.007
	RBias	0.093	0.117	0.094	0.138	0.070	0.080	0.085	0.108
	RMSE	0.086	0.012	0.090	0.011	0.063	0.008	0.076	0.010
	$\sqrt{\text{PEHE}}$	0.557	0.064	0.610	0.071	0.446	0.052	0.497	0.059
IE 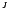	Bias	−0.126	0.018	−0.312	0.043	−0.097	0.012	−0.219	0.025
	RBias	0.069	0.074	0.169	0.176	0.051	0.053	0.114	0.106
	RMSE	0.177	0.023	0.394	0.049	0.133	0.016	0.274	0.031
	$\sqrt{\text{PEHE}}$	0.737	0.095	0.919	0.117	0.582	0.075	0.713	0.090
TE	Bias	−0.044	0.013	−0.090	0.010	−0.041	0.007	−0.083	0.006
	RBias	0.037	0.044	0.029	0.033	0.031	0.030	0.027	0.024
	RMSE	0.142	0.019	0.142	0.017	0.106	0.013	0.121	0.013
	$\sqrt{\text{PEHE}}$	0.824	0.110	0.921	0.120	0.646	0.087	0.717	0.096

Abbreviations: DE, the direct effect A→T; IE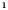 and IE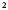, the separate indirect effect $A\to M_{1}\to T$ and $A\to M_{2}\to T$, respectively. IE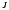, the joint indirect effect; Logh, the logarithm of hazards; Survp, survival probability; TE, the total effect.

In addition to setup (i), we conducted an additional simulation, referred to as setup (ii), to further validate the proposed model. This setup involved a fake mediator $M_{3}$ generated as a normal random variable from $N(-0.8x_{1}+1.2\sin(x_{7}-1)-(1+|x_{8}-x_{6}|A),1)$ and intentionally designed to have no effect on the survival outcome. The objective was to examine the impact of including the fake mediator, that is, the posttreatment variable that is listed among the candidate mediators but does not genuinely linking between the treatment and the survival outcome, on the estimation of the ICPSEs corresponding to both the truly existing causal pathways and the nonexistent one. Results summarized in Table S1 indicate that inclusion of the invalid mediator $M_{3}$ led to slightly increased average bias, RMSE, and $\sqrt{\text{PEHE}}$ for the estimated sample average interventional PSEs along the true underlying causal pathways. Nonetheless, the proposed model still demonstrated improved performance as the sample size increased. Moreover, the estimated indirect effect though $M_{3}$ separately was found consistently close to zero for each individual across the replications, suggesting that the incorporation of this invalid mediator did not induce any significant spurious effects into the discovered causal mechanism. This finding is also supported by Figure S2, which displays the distribution of the true and estimated ICPSEs along each causal pathway. Figure S3 shows the average posterior splitting proportions of the predictors, where the proposed method effectively captures the confounders and modifiers of the relationship between A and $M_{3}$, while simultaneously excludes $M_{3}$ from the selected predictors in the PH model.

To further evaluate the variable selection performance of the proposed method, we conducted additional simulations under scenarios where the number of redundant covariates or candidate mediators increased with the number of observations. Additionally, we performed sensitivity analyses with respect to different choices of hyperparameters, the number of spline basis functions, baseline hazard, and censoring rate. To demonstrate the robustness of the proposed method against violations of the normal assumption, we also considered non‐normally distributed mediators, where residual terms in model (2) were generated from heavy‐tailed, skewed, or mixture distributions. Performance of the proposed method was relatively stable across the scenarios. Detailed setups and results are provided in Appendix C of Supporting Information.

The computing code for conducting the preceding analysis is available at https://github.com/roxiesun/HMedCox.

## 6 Real Data Application

In this section, we applied the proposed method to a dataset extracted from the AIDS Clinical Trials Group Protocol 175 (ACTG175) [41] to demonstrate its utility. ACTG175 is a double‐blind clinical trial that collected medical history and demographic characteristics of 2139 adults infected with HIV to compare nucleoside monotherapy with zidovudine (ZDV) or didanosine (ddI) to combination therapy. Medical findings have suggested the superiority of combination therapy over monotherapy in slowing the progression of HIV disease, while recent advancements in causal inference revealed heterogeneity among patients of varying ages and sexual activity levels [8, 42].

**FIGURE 2:**
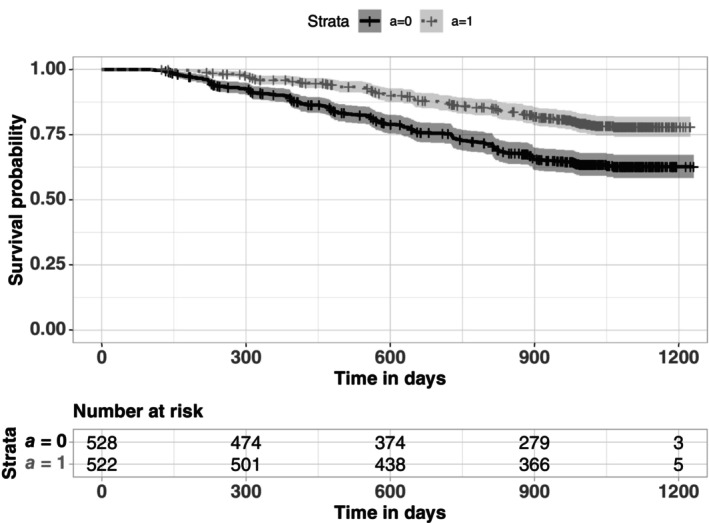
The Kaplan–Meier curves for HIV‐infected subjects who received zidovudine (ZDV)+didanosine (ddI) (A=1) and who received ZDV only (A=0) in the ACTG175 trial.

In this study, we focused on a subset of 532 subjects who received ZDV only as the control group (A=0) and 522 subjects who received ZDV+ddI combination as the treatment group (A=1). The primary objective is to identify the causal pathways linking combination therapy to the survival of individuals and explore potential sources of heterogeneity introduced by the effect modifiers. We considered three possible mediators: the CD4:CD8 ratio ($M_{1}$), as well as the changes in CD4 cell counts ($M_{2}$) and CD8 cell counts ($M_{3}$), all of which were measured at 20±5 weeks from baseline. Variation in the above two types of T cells are closely related to the low CD4:CD8 ratio for HIV‐infected individuals, which is indicative of the disease progression. The candidate mediators were regarded as causally non‐ordered based on two key considerations: first, they were measured simultaneously in a cross‐sectional manner, and secondly, the trends in these two types of cell counts are typically monitored parallelly in medical studies, along with their ratio, rather than being viewed as causally influencing one another. In the original study, the survival endpoint was defined as a decline in CD4 count of at least 50%, or occurrence of an AIDS‐defining event, or death. We excluded four individuals in the treatment group who had the event or were lost to follow‐up within the first 15 weeks after treatment initiation, resulting in a mean observed event time of 566 days, a maximum follow‐up time of 1,231 days, and a censoring rate of 73.3% for the targeted subset of individuals. Figure 2 depicts the Kaplan–Meier curves for subjects within the two treatment groups.

We considered 14 pretreatment covariates as potential confounders or effect modifiers, including six continuous variables: age (years), weight (kg), the number of days of previously received antiretroviral therapy (preanti, days), Karnofsky score (karnof, 0–100), baseline CD4 cell count (cd40, cells/mm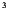), baseline CD8 cell count (cd80, cells/mm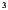), and eight binary variables: hemophilia (hemo, 1 = yes), homosexual activity (homo, 1 = yes), history of intravenous drug use (drugs, 1 = yes), ZDV use in the 30 days prior to treatment initiation (z30, 1 = yes), race (0 = White, 1 = non‐White), gender (1 = male), history of prior antiretroviral therapy (str2, 1 = experienced), and symptoms of HIV infection at enrolment (symptom, 0 = asymptomatic, 1 = symptomatic). The estimated propensity score was also included as a predictor.

**FIGURE 3:**
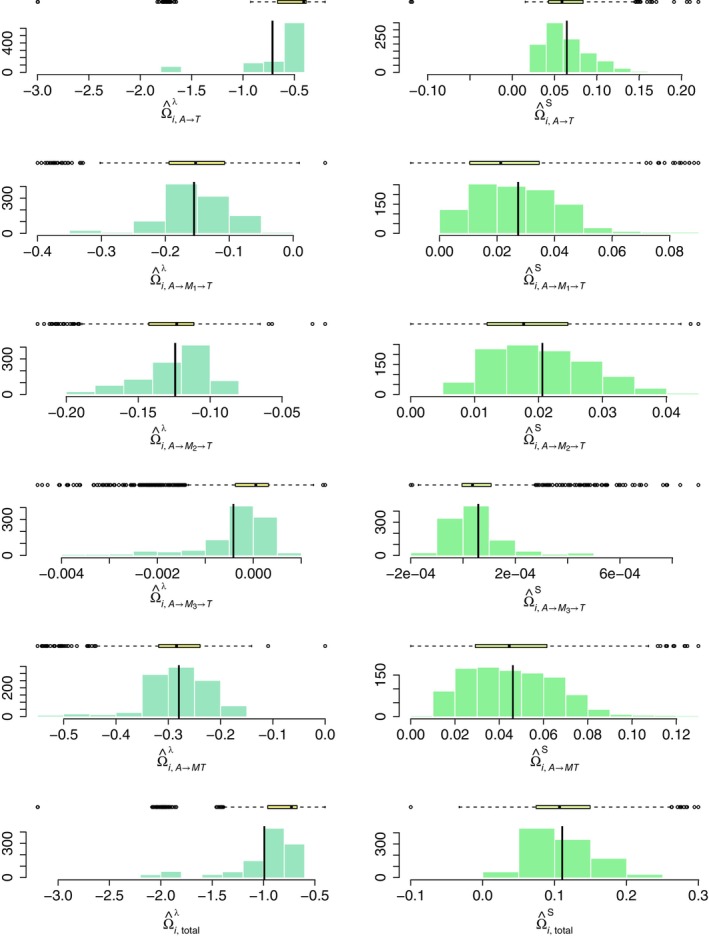
Estimated ICPSEs for each individual on the logarithm scale of hazards (left) and survival probability with respect to t=566 days (right). The vertical black lines stand for the estimated sample average interventional PSEs correspondingly.

We used the trace plots of three Markov chains starting from different initial values to check convergence. Figure S4 depicts the trace plots of several randomly selected parameters, showing that the Markov chains mixed well within a few iterations. Therefore, we ran 5000 MCMC iterations and discarded the first half as burn‐in. The upper panel of Table 2 presents the estimated sample average interventional PSEs and the proportion of individuals with various levels of differential ICPSEs based on the posterior samples. The combination therapy of ZDV+ddI shows an advantage over ZDV only in slowing the progression of HIV infection with an average hazard ratio of exp(−0.994)=0.370 and an average increase of 0.110 in the probability of surviving over 566 days. Specifically, we observed that the treatment effect is mainly carried out through the direct causal pathway and partially through the first two mediators, that is, the CD4:CD8 ratio and changes in the CD4 cell counts measured at 20±5 weeks. In contrast, the changes in CD8 cell counts appeared to be an invalid mediator. The estimated sample average indirect effect that corresponds to the $A\to M_{3}\to T$ pathway is nonsignificant and consistently near zero on both scales, suggesting that $M_{3}$ does not truly mediate the effect of the combination therapy on survival of the HIV‐infected subjects. On the scale of the logarithm of hazard, we spotted only mild evidence of heterogeneity on the direct pathway, with 13.7% of the subjects exhibiting a differential direct effect with $D_{i,A\to T}^{⋆}>0.7$. On the probability of surviving over 566 days, however, such evidences were detected on both the direct pathway and the indirect pathways through $M_{1}$ and $M_{2}$. Figure 3 shows the boxplot and histogram of the estimated ICPSEs across each causal pathway.

**TABLE 2:** Estimated sample average interventional path‐specific effects (PSEs) on the scale of logarithm of hazard and survival probability, together with the evidence of individual‐specific differential PSEs and the top selected splitting variables in the analysis of ACTG175.

		Scale		
Path	Index	Logh		Survp
DE	Est.(SD)	−0.714(0.303)		0.064 (0.020)
	$D_{i,A\to T}^{*}>0.9$	0%		1.9%
	$D_{i,A\to T}^{*}>0.8$	0%		7.1%
	$D_{i,A\to T}^{*}>0.7$	13.7%		15.9%
IE 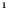	Est. (SD)	−0.155(0.075)		0.027 (0.013)
	$D_{i,A\to M_{1}\to T}^{*}>0.9$	0%		0.2%
	$D_{i,A\to M_{1}\to T}^{*}>0.8$	0%		5.0%
	$D_{i,A\to M_{1}\to T}^{*}>0.7$	0.1%		12.2%
IE 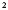	Est. (SD)	−0.125(0.113)		0.021 (0.019)
	$D_{i,A\to M_{2}\to T}^{*}>0.9$	0%		0%
	$D_{i,A\to M_{2}\to T}^{*}>0.8$	0%		0.5%
	$D_{i,A\to M_{2}\to T}^{*}>0.7$	0%		15.0%
IE 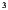	Est. (SD)	−0.001(0.003)		0.000 (0.001)
	$D_{i,A\to M_{3}\to T}^{*}>0.9$	0%		0%
	$D_{i,A\to M_{3}\to T}^{*}>0.8$	0%		0%
	$D_{i,A\to M_{3}\to T}^{*}>0.7$	0%		0%
IE 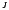	Est. (SD)	−0.280(0.131)		0.046 (0.021)
	$D_{i,A\to MT}^{*}>0.9$	0%		0.1%
	$D_{i,A\to MT}^{*}>0.8$	0%		2.3%
	$D_{i,A\to MT}^{*}>0.7$	0.1%		8.2%
TE	Est. (SD)	−0.994(0.305)		0.110 (0.023)
	$D_{i,total}^{*}>0.9$	0%		1.4%
	$D_{i,total}^{*}>0.8$	0%		5.9%
	$D_{i,total}^{*}>0.7$	1.0%		13.0%
		**Tree ensembles**		
**Top splitting variable**		$\upsilon_{M}$	υ	$\tau \&\tau_{M}$
First		Gender (17.4%)	$M_{1}$ (11.9%)	Homo (34.2%)
Second		Homo (17.2%)	Drugs (8.3%)	Hemo (32.4%)
Third		Race (17.0%)	Race (8.3%)	Drugs (31.5%)
Fourth		cd80 (12.3%)	Karnof (8.3%)	Weight (0.9%)

Abbreviations: Est, Bayesian estimates of the sample average PSEs; SD, posterior standard deviation.

**FIGURE 4:**
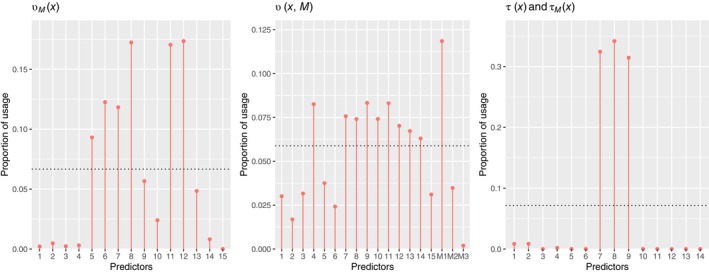
Posterior splitting proportions in the tree ensembles for each covariate in the ACTG175 dataset. The horizontal dotted lines stand for the discrete uniform splitting probabilities.

The lower panel of Table 2 presents the top selected splitting variables for each component of the tree ensembles, which serve as the most possible sources of heterogeneity. The posterior splitting proportions for the predictors in each tree ensemble are plotted in Figure 4. Overall, the effects of the combination therapy on the mediators and the survival outcomes are moderated by hemophilia, intravenous drug use history, and homosexual activity. Figure S5 depicts the partial effects of the effect modifiers on the distribution of the ICPSEs. Finally, the levels of the mediators at 20±5 weeks are mainly related to gender, homosexual activity, race, and the baseline CD8 cell counts; while the prognosis of the HIV‐infected subjects can also be confounded by the Karnofsky score, race, and homosexual activity.

## 7 Conclusion

This study introduces a novel heterogeneous mediation analysis tailored for survival data with multiple potential mediators based on joint modeling of the mediator regression model and the PH model. The proposed approach with shared ensemble of trees explicitly accounts for the overlapped pattern of confounders and effect modifiers among the mediators and outcome, which are ubiquitous in real‐world scenarios. The incorporation of sparsity‐inducing Gibbs‐type priors into the shared tree ensembles addresses the challenges of feature selection and heterogeneity quantification, enabling the model to identify the most relevant predictors while simultaneously capturing the intricate interplay between effect modifiers across multiple causal pathways. A fully Bayesian approach is developed to estimate the individual‐specific and sample average interventional PSEs under the potential outcome framework. The utility and performance of the proposed method are thoroughly evaluated through extensive simulation studies and a real‐world application to the ACTG175 dataset.

There are several promising new directions that can be explored. First, we have assumed causally non‐ordered mediators to model their joint distribution with the time‐to‐event outcome through three separate ensembles of trees. The marginal distribution of each mediator is therefore directly attainable to facilitate the Monte Carlo implementation of the mediator formula. Strengths of the shared tree topologies are manifested in contrast to modeling the prognostic functions and direct causal pathways separately for the mediator and outcome regression models using $2(p_{m}+1)$ tree ensembles with univariate leaf node parameters. However, it is worth mentioning that such efficiency of shared structure may trade off the ability to disentangle the causal structure among multiple causally ordered mediators. A possible solution would be to substitute $\upsilon_{M}(x_{i})$ in Equation (2) with $\upsilon_{M}(x_{i},M_{i})$, allowing each mediator to be regressed on the rest of mediators (excluding itself), and embedding the relevant field knowledge on their causal ordering through prior distributions assigned to the corresponding splitting rules of the tree ensembles. We consider this as a promising future direction that warrants continuing effort, given that the causal structure learning problem itself remains an ongoing topic of debate. From the perspective of variable selection, it is also a worthwhile endeavor to allow p and $p_{m}$ to be high‐dimensional and study the respective asymptotic properties regarding their order. Second, the interventionist framework employed in this study can handle more general cases, such as the presence of posttreatment confounding, without the need to impose the cross‐world independence assumption when focusing on the sample average or population level effects. Although our proposed method can accommodate posttreatment variables as possible mediators, incorporating heterogeneity and addressing more general causal structure simultaneously remains a direction for future research. Therefore, relaxing this assumption under the current joint model is of considerable interest. Third, it should also be possible to address violations of the positivity assumption, which can be viewed as an extreme case of heterogeneity. Exploring the above extensions would require substantial efforts in future research endeavors.

## Conflicts of Interest

The authors declare no conflicts of interest.

## Supporting information




**Data S1.** Supporting information.

## Data Availability

Data sharing is not applicable to this article as no new data were created or analyzed in this study.
