# Supplementary material for: Heterogeneous Mediation Analysis for Cox Proportional Hazards Model With Multiple Mediators
Source: Stat Med. 2024 Oct 28;43(29):5497–512. doi: 10.1002/sim.10239 (PMC11588993; doi:10.1002/sim.10239)
Supplement: Supplementary file 1 — Data S1. Supporting information. [file SIM-43-5497-s001.pdf]

Supplementary material for "Heterogeneous mediation analysis for Cox proportional hazards model with multiple mediators" by Rongqian Sun and Xinyuan Song

Web Appendix A   Derivation of Equation (13)-(16)

Under the identification assumptions stated in Section 3, the targeted function of potential survival time for subject i, $\omega\left\{T_i\big(a, \acute{M}_{i1}(a_1), \ldots, \acute{M}_{i,p_m}(a_{p_m})\big)\right\}$, is identified as

$$
\begin{aligned}
&\omega\left\{T_i\big(a, \acute{M}_{i1}(a_1), \ldots, \acute{M}_{i,p_m}(a_{p_m})\big)\right\} \\
=&\int\cdots\int \omega\left\{T_i\big(a, m_1, \ldots, m_{p_m}\big) \mid \mathbf{x}_i = \boldsymbol{x}\right\} dF_{M_{i1}(a_1)|\mathbf{x}_i}(m_1)\cdots dF_{M_{i,p_m}(a_{p_m})|\mathbf{x}_i}(m_{p_m}) \\
&\text{[By Assumption 3]} \\
=&\int\cdots\int \omega\left\{T_i\big(a, m_1, \ldots, m_{p_m}\big) \mid \mathbf{x}_i = \boldsymbol{x}\right\} dF_{M_{i1}(a_1)|A_i=a_1,\mathbf{x}_i}(m_1)\cdots dF_{M_{i,p_m}(a_{p_m})|A_i=a_{p_m},\mathbf{x}_i}(m_{p_m}) \\
&\text{[By Consistency]} \\
=&\int\cdots\int \omega\left\{T_i\big(a, m_1, \ldots, m_{p_m}\big) \mid \mathbf{x}_i = \boldsymbol{x}\right\} dF_{M_{i1}|A_i=a_1,\mathbf{x}_i}(m_1)\cdots dF_{M_{i,p_m}|A_i=a_{p_m},\mathbf{x}_i}(m_{p_m}) \\
&\text{[By Assumption 1]} \\
=&\int\cdots\int \omega\left\{T_i\big(a, m_1, \ldots, m_{p_m}\big) \mid A_i = a, \mathbf{x}_i = \boldsymbol{x}\right\} dF_{M_{i1}|A_i=a_1,\mathbf{x}_i}(m_1)\cdots dF_{M_{i,p_m}|A_i=a_{p_m},\mathbf{x}_i}(m_{p_m}) \\
&\text{[By Assumption 2]} \\
=&\int\cdots\int \omega\left\{T_i\big(a, m_1, \ldots, m_{p_m}\big) \mid A_i = a, M_{i1} = m_1, \ldots, M_{i,p_m} = m_{p_m}, \mathbf{x}_i = \boldsymbol{x}\right\} dF_{M_{i1}|A_i=a_1,\mathbf{x}_i}(m_1)\cdots dF_{M_{i,p_m}|A_i=a_{p_m},\mathbf{x}_i}(m \text{ [illegible]} \\
&\text{[By Consistency]} \\
=&\int\cdots\int \omega\left\{T_i \mid A_i = a, M_{i1} = m_1, \ldots, M_{i,p_m} = m_{p_m}, \mathbf{x}_i = \boldsymbol{x}\right\} dF_{M_{i1}|A_i=a_1,\mathbf{x}_i}(m_1)\cdots dF_{M_{i,p_m}|A_i=a_{p_m},\mathbf{x}_i}(m_{p_m})
\end{aligned}
\tag{S.1}
$$

The last equation follows from the basic consistency assumption of the counterfactual framework, which states that an individual's potential outcome under their observed exposure history is exactly their observed outcome[1,2], i.e., $M_{iq}(A_i) = M_{iq}$ and $T_i(A_i, \mathbf{M}_i(A_i)) = T_i$.

$\omega\left\{T_i\big(a, \{\acute{M}_{i1}(a'), \ldots, \acute{M}_{i,p_m}(a')\}\big)\right\}$ can be identified in a similarly way as

$$\begin{aligned}
&\omega\left\{T_i\big(a, \{\acute{M}_{i1}(a'), \ldots, \acute{M}_{i,p_m}(a')\}\big)\right\} \\
&= \int \omega\left\{T_i\big(a, m_1, \ldots, m_{p_m}\big) \mid \mathbf{x}_i = \boldsymbol{x}\right\} dF_{M_{i1}(a'),\ldots,M_{ip_m}(a')|\mathbf{x}_i}(m_1, \ldots, m_{p_m}), \\
&= \int \omega\left\{T_i\big(a, m_1, \ldots, m_{p_m}\big) \mid \mathbf{x}_i = \boldsymbol{x}\right\} dF_{M_{i1}(a'),\ldots,M_{ip_m}(a')|A_i=a',\mathbf{x}_i}(m_1, \ldots, m_{p_m}), \\
&= \int \omega\{T_i\big(a, m_1, \ldots, m_{p_m}\big) \mid \mathbf{x}_i = \boldsymbol{x}\} dF_{\mathbf{M}_i|A_i=a',\mathbf{x}_i=\boldsymbol{x}}(m_1, \ldots, m_{p_m}), \\
&= \int \omega\{T_i\big(a, m_1, \ldots, m_{p_m}\big) \mid A_i = a, \mathbf{x}_i = \boldsymbol{x}\} dF_{\mathbf{M}_i|A_i=a',\mathbf{x}_i=\boldsymbol{x}}(m_1, \ldots, m_{p_m}), \\
&= \int \omega\{T_i\big(a, m_1, \ldots, m_{p_m}\big) \mid A_i = a, M_{i1} = m_1, \ldots, M_{i,p_m} = m_{p_m}, \mathbf{x}_i = \boldsymbol{x}\} dF_{\mathbf{M}_i|A_i=a',\mathbf{x}_i=\boldsymbol{x}}(m_1, \ldots, m_{p_m}), \\
&= \int \omega\left\{T_i \mid A_i = a, M_{i1} = m_1, \ldots, M_{i,p_m} = m_{p_m}, \mathbf{x}_i = \boldsymbol{x}\right\} dF_{\mathbf{M}_i|A_i=a',\mathbf{x}_i=\boldsymbol{x}}(m_1, \ldots, m_{p_m}).
\end{aligned} \tag{S.2}$$

We used the Monte Carlo method to integrate over $F_{M_{iq}|A_i,\mathbf{x}_i}$ or $F_{\mathbf{M}_i|A_i,\mathbf{x}_i}$, and also adopted the comonotonic sampling strategy[3] to enhance the efficiency of the numerical implementation of the mediation formula. For a simple single-mediator case with $p_m = 1$, $M_i^{(itr)}(1)$ and $M_i^{(itr)}(0)$ are sampled as comonotonic pairs using inverse transform sampling with shared uniform samples u_{ik}. This approach is effective in reducing Monte Carlo errors by making the $M_i^{(itr)}(a)$ pairs as correlated as possible, thereby allowing for accurate estimation of the ICPSEs with only one sample (i.e., $K_M = 1$) per iteration. In multiple-mediator cases with $p_m \geq 2$, the Monte Carlo implementation of the mediation formula can be achieved efficiently through copula, which can be viewed as the multivariate analogy of the comonotonic inverse transformation sampling. The major steps are provided in Algorithm S1.

The group-average or sample-average interventional PSEs are identified through an additional integration over the covariate distribution $F_{\mathbf{x}_i}$. We followed the common practice in the mediation literature to approximate the integration using empirical distribution of the covariates based on the collected sample[4,5], under which circumstance Equation (15)

Algorithm S1: The Monte Carlo implementation of mediation formula with $p_m \geq 2$

Input: $\{\mathcal{T}_j^{(itr)}, \boldsymbol{\mathcal{M}}_j^{(itr)}\}_{j=1}^J$, $\{\widetilde{\mathcal{T}}_h^{(itr)}, \widetilde{\boldsymbol{\mathcal{M}}}_h^{(M,itr)}, \widetilde{\mathcal{M}}_h^{(itr)}\}_{h=1}^H$, $\{\breve{\mathcal{T}}_k, \breve{\mathcal{M}}_k\}_{k=1}^K$, $\boldsymbol{\gamma}^{(itr)}$, $\boldsymbol{\Sigma}^{(itr)}$, at iteration itr.

Output: the interventional conditional path-specific effects (ICPSEs).

for $i = 1, \ldots, n$ **do**

  for $k = 1, \ldots, K_M$ **do**

    Sample $\mathbf{M}_i^{(itr,k)}(1) = \left(M_{i1}^{(itr,k)}(1), M_{i2}^{(itr,k)}(1), \ldots, M_{i,p_m}^{(itr,k)}(1)\right)^T \sim N\left(\boldsymbol{\upsilon}_M^{(itr)}(\mathbf{x}_i) + \boldsymbol{\tau}_M^{(itr)}(\mathbf{x}_i), \boldsymbol{\Sigma}^{(itr)}\right)$;

    for $q = 1, \ldots, p_m$ **do**

      Compute $u_{iqk} = F_{M_q|A_i=1,\mathbf{x}_i}\left(M_{iq}^{(itr,k)}(1) \mid \{\mathcal{T}_j^{(itr)}, \boldsymbol{\mathcal{M}}_j^{(itr,k)}\}_{j=1}^J, \{\widetilde{\mathcal{T}}_h^{(itr)}, \widetilde{\boldsymbol{\mathcal{M}}}_h^{(M,itr)}\}_{h=1}^H, \boldsymbol{\Sigma}^{(itr)}\right)$;

      Sample $M_{iq}^{(itr,k)}(0) = F^{-1}_{M_q|A_i=0,\mathbf{x}_i}\left(u_{iqk} \mid \{\mathcal{T}_j^{(itr)}, \boldsymbol{\mathcal{M}}_j^{(itr)}\}_{j=1}^J, \{\widetilde{\mathcal{T}}_h^{(itr)}, \widetilde{\boldsymbol{\mathcal{M}}}_h^{(M,itr)}\}_{h=1}^H, \boldsymbol{\Sigma}^{(itr)}\right)$;

  Compute the individual-specific ICPSEs:

$$\Omega_{i,A\to T}^{(itr)}(1) = \frac{1}{K_M}\sum_{k=1}^{K_M} \omega\left\{T_i\big(1, \{M_{i1}^{(itr,k)}(1), \ldots, M_{i,p_m}^{(itr,k)}(1)\}\big)\right\} - \omega\left\{T_i\big(0, \{M_{i1}^{(itr,k)}(1), \ldots, M_{i,p_m}^{(itr,k)}(1)\}\big)\right\},$$

$$\Omega_{i,A\to\mathbf{M}T}^{(itr)}(0) = \frac{1}{K_M}\sum_{k=1}^{K_M} \omega\left\{T_i\big(0, \{M_{i1}^{(itr,k)}(1), \ldots, M_{i,p_m}^{(itr,k)}(1)\}\big)\right\} - \omega\left\{T_i\big(0, \{M_{i1}^{(itr,k)}(0), \ldots, M_{i,p_m}^{(itr,k)}(0)\}\big)\right\}$$

$$\Omega_{i,A\to M_q\to T}^{(itr)}(0) = \frac{1}{K_M}\sum_{k=1}^{K_M} \omega\left\{T_i\big(0, M_{i1}^{(itr,k)}(0), \ldots, M_{i,q-1}^{(itr,k)}(0), M_{iq}^{(itr,k)}(1), M_{i,q+1}^{(itr,k)}(0), \ldots, M_{i,p_m}^{(itr,k)}(0)\big)\right\} - \omega\left\{T_i\big(0, M_{i1}^{(itr,k)}(0), \ldots, M_{i,p_m}^{(itr,k)}(0)\big)\right\},\ q = 1, \ldots, p_m,$$

$$\Omega_{i,total}^{(itr)} = \frac{1}{K_M}\sum_{k=1}^{K_M} \omega\left\{T_i\big(1, \{M_{i1}^{(itr,k)}(1), \ldots, M_{i,p_m}^{(itr,k)}(1)\}\big)\right\} - \omega\left\{T_i\big(0, \{M_{i1}^{(itr,k)}(0), \ldots, M_{i,p_m}^{(itr,k)}(0)\}\big)\right\},$$

  on each scale numerically based on Equations (16)-(20);

Compute the sample average interventional PSEs: $\bar{\Omega}_{A\to T}(1) = 1/n\sum_{i=1}^n \Omega_{i,A\to T}^{(itr)}(1)$, $\bar{\Omega}_{A\to\mathbf{M}T}(0) = 1/n\sum_{i=1}^n \Omega_{i,A\to\mathbf{M}T}^{(itr)}(0)$, $\bar{\Omega}_{A\to M_q\to T}^{(itr)}(0) = \frac{1}{n}\sum_{i=1}^n \Omega_{i,A\to M_q\to T}^{(itr)}(0)$, and $\bar{\Omega}_{total}^{(itr)} = 1/n\sum_{i=1}^n \Omega_{i,total}^{(itr)}$, on each scale.

simplifies to

$$
\begin{aligned}
&\bar{\omega}\left\{T_i\big(a, \acute{M}_{i1}(a_1), \ldots, \acute{M}_{i,p_m}(a_{p_m})\big)\right\} \\
\approx&\frac{1}{n}\sum_{i=1}^{n}\omega\left\{T_i\big(a, \acute{M}_{i1}(a_1), \ldots, \acute{M}_{i,p_m}(a_{p_m})\big)\right\} \\
=&\frac{1}{n}\sum_{i=1}^{n}\iiint \omega\left\{T_i \mid A_i = a, M_{i1} = m_1, \ldots, M_{i,p_m} = m_{p_m}, \mathbf{x}_i = \boldsymbol{x}\right\} dF_{M_{i1}|A_i=a_1,\mathbf{x}_i}(m_1)\cdots dF_{M_{i,p_m}|A_i=a_{p_m},\mathbf{x}_i}(m_{p_m})
\end{aligned} \tag{S.3}
$$

and Equation (16) to

$$
\begin{aligned}
&\bar{\omega}\left\{T_i\big(a, \{\acute{M}_{i1}(a'), \ldots, \acute{M}_{i,p_m}(a')\}\big)\right\} \\
\approx&\frac{1}{n}\sum_{i=1}^{n}\omega\left\{T_i\big(a, \{\acute{M}_{i1}(a'), \ldots, \acute{M}_{i,p_m}(a')\}\big)\right\} \\
=&\frac{1}{n}\sum_{i=1}^{n}\int\int \omega\left\{T_i \mid A_i = a, M_{i1} = m_1, \ldots, M_{i,p_m} = m_{p_m}, \mathbf{x}_i = \boldsymbol{x}\right\} dF_{\mathbf{M}_i|A_i=a',\mathbf{x}_i=\boldsymbol{x}}(m_1, \ldots, m_{p_m}).
\end{aligned} \tag{S.4}
$$

Web Appendix B Full conditional distributions of the tree ensembles

Denote $\mathbf{Y} = (Y_1, Y_2, \ldots, Y_n)^T$, $\mathfrak{M} = (\mathbf{M}_1, \mathbf{M}_2, \ldots, \mathbf{M}_n)^T$, $\mathbf{A} = (A_1, A_2, \ldots, A_n)^T$, $\mathbf{X} = (\mathbf{x}_1^T, \mathbf{x}_2^T, \ldots, \mathbf{x}_n^T)^T$, and $\boldsymbol{\delta} = (\delta_1, \ldots, \delta_n)^T$. Let $\mathcal{D} = \{\mathbf{Y}, \mathbf{X}, \mathbf{A}, \boldsymbol{\delta}, \mathfrak{M}\}$ denote the observed data. The complete data likelihood of the proposed model is expressed as

$$
\begin{aligned}
p(\mathcal{D}|\cdot) = &\prod_{i=1}^{n}\exp\big[-\Lambda_0(Y_i)\exp\{\upsilon(\mathbf{x}_i, \mathbf{M}_i) + \tau(\mathbf{x}_i)A_i\}\big]\big[\lambda_0(Y_i)\exp\{\upsilon(\mathbf{x}_i, \mathbf{M}_i) + \tau(\mathbf{x}_i)A_i\}\big]^{\delta_i} \\
&\times (2\pi)^{-p_m/2}\det(\boldsymbol{\Sigma})^{-1/2}\exp\left\{-\frac{1}{2}\left(\mathbf{M}_i - \boldsymbol{\upsilon}_M(\mathbf{x}_i) - \boldsymbol{\tau}_M(\mathbf{x}_i)A_i\right)^T\boldsymbol{\Sigma}^{-1}\left(\mathbf{M}_i - \boldsymbol{\upsilon}_M(\mathbf{x}_i) - \boldsymbol{\tau}_M(\mathbf{x}_i)A_i\right)\right\}
\end{aligned} \tag{S.5}
$$

where $\Lambda_0(t) = \int_0^t \lambda_0(u)du$ is the cumulative baseline hazard function. After assuming prior independence among the unknown parameters $\{\mathcal{T}_j, \boldsymbol{\mathcal{M}}_j\}_{j=1}^{J}$, $\{\widetilde{\mathcal{T}}_h, \widetilde{\boldsymbol{\mathcal{M}}}_h^{(M)}, \widetilde{\mathcal{M}}_h\}_{h=1}^{H}$, $\{\breve{\mathcal{T}}_k, \breve{\mathcal{M}}_j\}_{k=1}^{K}$,

$\boldsymbol{\gamma}$, and $\boldsymbol{\Sigma}$, and prior independence among the individual binary trees inside each tree ensembles as well, the full conditional distribution of the parameters are derived as follows.

(I) **Full conditional distribution of** $\{\mathcal{T}_j, \boldsymbol{\mathcal{M}}_j\}_{j=1}^J$

Let $\boldsymbol{R}_{ij} = \mathbf{M}_i - \sum_{l\neq j} g_1(\mathbf{x}_i; \mathcal{T}_l, \boldsymbol{\mathcal{M}}_l) - \boldsymbol{\tau}_M(\mathbf{x}_i)A_i$ and $\boldsymbol{R}_j = (\boldsymbol{R}_{1j}, \ldots, \boldsymbol{R}_{nj})^T$ denote the partial residuals in the mediator regression equation w.r.t the jth binary tree $\{\mathcal{T}_j, \boldsymbol{\mathcal{M}}_j\}$. By prior independence and the i.i.d. assumption on the sample data,

$$p\left(\{\mathcal{T}_j, \boldsymbol{\mathcal{M}}_j\}_{j=1}^J | \mathcal{D}, \boldsymbol{\Sigma}, \{\widetilde{\mathcal{T}}_h, \widetilde{\boldsymbol{\mathcal{M}}}_h^{(M)}\}_{h=1}^H\right) = \prod_{j=1}^J p(\boldsymbol{\mathcal{M}}_j | \boldsymbol{R}_j, \mathcal{T}_j, \boldsymbol{\Sigma}) p(\mathcal{T}_j | \boldsymbol{R}_j, \boldsymbol{\Sigma}),$$

where

$$\begin{aligned} p(\boldsymbol{\mathcal{M}}_j | \mathcal{D}, \mathcal{T}_j, \boldsymbol{\Sigma}) &\propto p(\boldsymbol{R}_j | \boldsymbol{\mathcal{M}}_j, \mathcal{T}_j, \boldsymbol{\Sigma}) p(\boldsymbol{\mathcal{M}}_j | \mathcal{T}_j) \\ &\propto \prod_{l=1}^{b_j} \prod_{i:\mathbb{1}_{il}=1} \exp\Big\{ -\frac{1}{2}(\boldsymbol{R}_{ij} - \boldsymbol{\mu}_{jl})^T \boldsymbol{\Sigma}^{-1} (\boldsymbol{R}_{ij} - \boldsymbol{\mu}_{jl})\Big\} \exp\Big\{ -\frac{1}{2}\boldsymbol{\mu}_{jl}^T \boldsymbol{\Sigma}_\mu^{-1} \boldsymbol{\mu}_{jl}\Big\} \\ &\propto \prod_{l=1}^{b_j} \exp\Big\{ -\frac{1}{2}\boldsymbol{\mu}_{jl}^T (\sum_{i:\mathbb{1}_{il}=1} \boldsymbol{\Sigma}^{-1} + \boldsymbol{\Sigma}_\mu^{-1}) \boldsymbol{\mu}_{jl} - \sum_{i:\mathbb{1}_{il}=1} \boldsymbol{R}_{ij}^T \boldsymbol{\Sigma}^{-1} \boldsymbol{\mu}_{jl} \Big\} \\ &\stackrel{d}{=} \prod_{l=1}^{b_j} N(\boldsymbol{a}_l, \boldsymbol{\Sigma}_l), \end{aligned} \tag{S.6}$$

with $\boldsymbol{\Sigma}_l = (n_l \boldsymbol{\Sigma}^{-1} + \boldsymbol{\Sigma}_\mu^{-1})^{-1}$, $\boldsymbol{a}_l = \sum_{i:\mathbb{1}_{il}=1} \boldsymbol{R}_{ij}^T \boldsymbol{\Sigma}^{-1} (n_l \boldsymbol{\Sigma}^{-1} + \boldsymbol{\Sigma}_\mu^{-1})^{-1}$, $\boldsymbol{\Sigma}_\mu = \frac{c_1^2}{4Jk_1^2}\boldsymbol{\Sigma}_0$, $\mathbb{1}_{il}$ denoting that subject i is allocated to the lth terminal node of $\mathcal{T}_j$ with mean parameter μ_{jl}, $n_l = \sum_i \mathbb{1}_{il}$, and '$\stackrel{d}{=}$' representing equivalence in distribution. The conjugacy makes it feasible to draw $\mathcal{T}_j$ from its marginal posterior distribution where $\boldsymbol{\mathcal{M}}_j$ is integrated out to

avoid the need for reversible jumps. That is,

$$
\begin{aligned}
p(\mathcal{T}_j|\boldsymbol{R}_j,\boldsymbol{\Sigma}) &\propto p(\mathcal{T}_j)\left(\int p(\boldsymbol{R}_j|\boldsymbol{\mathcal{M}}_j,\mathcal{T}_j,\boldsymbol{\Sigma})p(\boldsymbol{\mathcal{M}}_j|\mathcal{T}_j)d\boldsymbol{\mathcal{M}}_j\right)\\
&\propto p(\mathcal{T}_j)\prod_{l=1}^{b_j}\int\prod_{i:\mathbb{1}_{il}=1}\exp\Big\{-\frac{1}{2}(\boldsymbol{R}_{ij}-\boldsymbol{\mu}_{jl})^T\boldsymbol{\Sigma}^{-1}(\boldsymbol{R}_{ij}-\boldsymbol{\mu}_{jl})\Big\}\exp\Big\{-\frac{1}{2}\boldsymbol{\mu}_{jl}^T\boldsymbol{\Sigma}_{\mu}^{-1}\boldsymbol{\mu}_{jl}\Big\}d\boldsymbol{\mu}_{jl}\\
&\propto p(\mathcal{T}_j)\prod_{l=1}^{b_j}\left(\frac{1}{\sqrt{2\pi}}\right)^{n_lp_m}\det(\boldsymbol{\Sigma})^{(-n_l/2)}\det(\boldsymbol{\Sigma}_{\mu})^{(-1/2)}\det(\boldsymbol{\Sigma}_l)^{1/2}\exp\left\{-\frac{1}{2}\sum_{i:\mathbb{1}_{il}=1}\boldsymbol{R}_{ij}^T\boldsymbol{\Sigma}^{-1}\boldsymbol{R}_{ij}\right\}\\
&\times\exp\left\{\frac{1}{2}\left(\sum_{i:\mathbb{1}_{il}=1}\boldsymbol{R}_{ij}\right)^T\boldsymbol{\Sigma}^{-1}\boldsymbol{\Sigma}_l\boldsymbol{\Sigma}^{-1}\left(\sum_{i:\mathbb{1}_{il}=1}\boldsymbol{R}_{ij}\right)\right\}.\\
&\propto p(\mathcal{T}_j)\left(\frac{1}{\sqrt{2\pi}}\right)^{np_m}\det(\boldsymbol{\Sigma})^{(-n/2)}\exp\left\{-\frac{1}{2}\sum_{i=1}^{n}\boldsymbol{R}_{ij}^T\boldsymbol{\Sigma}^{-1}\boldsymbol{R}_{ij}\right\}\prod_{l=1}^{b_j}\det(\boldsymbol{\Sigma}_{\mu})^{(-1/2)}\det(\boldsymbol{\Sigma}_l)^{1/2}\\
&\times\exp\left\{\frac{1}{2}\left(\sum_{i:\mathbb{1}_{il}=1}\boldsymbol{R}_{ij}\right)^T\boldsymbol{\Sigma}^{-1}\boldsymbol{\Sigma}_l\boldsymbol{\Sigma}^{-1}\left(\sum_{i:\mathbb{1}_{il}=1}\boldsymbol{R}_{ij}\right)\right\}.
\end{aligned}
\tag{S.7}
$$

To acquire posterior samples of $\mathcal{T}_j$, we employed the Metropolis-Hastings algorithm[6,7], with a proposal distribution suggested by Chipman et al.[8]. This proposal distribution is a mixture of local modifications to the current iteration $\mathcal{T}_j^{(itr)}$ that includes three possible moves with probabilities as follows: splitting at a leaf node (0.35), pruning a pair of leaf nodes (0.35), and changing the splitting rule of an internal node (0.3). A fourth moves suggested by Linero and Du[9] was also considered, which simply generates a candidate tree from the prior distribution of $\mathcal{T}_j$ conditional on $(\mathcal{T}_{-j},\mathcal{M}_{-j})$. Given the current iteration $\mathcal{T}_j^{(itr)}$, the candidate tree $\mathcal{T}_j^*$ is proposed according to the proposal distribution $q(\cdot|\cdot)$, and is accepted as $\mathcal{T}_j^{(itr+1)}$ with a probability of $\min\left\{1,\frac{q\left(\mathcal{T}_j^{(itr)}\middle|\mathcal{T}_j^*\right)p\left(\mathcal{T}_j^*\middle|\boldsymbol{R}_j^{(itr)},\boldsymbol{\Sigma}^{(itr)}\right)}{q\left(\mathcal{T}_j^*\middle|\mathcal{T}_j^{(itr)}\right)p\left(\mathcal{T}_j^{(itr)}\middle|\boldsymbol{R}_j^{(itr)},\boldsymbol{\Sigma}^{(itr)}\right)}\right\}$.

(II) **Full conditional distribution of** $\{\widetilde{\mathcal{T}}_h,\widetilde{\mathcal{M}}_h,\widetilde{\boldsymbol{\mathcal{M}}}_h^{(M)}\}_{h=1}^{H}$

Let $\widetilde{\boldsymbol{R}}_{ih}^{(M)}=\mathbf{M}_i-\boldsymbol{\upsilon}_M(\mathbf{x}_i)-\sum_{l\neq h}g_2(\mathbf{x}_i;\widetilde{\mathcal{T}}_l,\widetilde{\boldsymbol{\mathcal{M}}}_l^{(M)})A_i$ and $\widetilde{\boldsymbol{R}}_h^{(M)}=(\widetilde{\boldsymbol{R}}_{1h}^{(M)},\ldots,\widetilde{\boldsymbol{R}}_{nh}^{(M)})^T$ denote the partial residuals in the mediator regression equation w.r.t the hth binary tree $\{\widetilde{\mathcal{T}}_h,\widetilde{\boldsymbol{\mathcal{M}}}_h^{(M)}\}$. Let $\widetilde{R}_{ih}=\upsilon(\mathbf{x}_i,\mathbf{M}_i)+\sum_{l\neq h}g_2(\mathbf{x}_i;\widetilde{\mathcal{T}}_l,\widetilde{\mathcal{M}}_l)A_i$ and $\widetilde{\boldsymbol{R}}_h=(\widetilde{R}_{1h},\ldots,\widetilde{R}_{nh})^T$ denote

the partial residuals in the PH model w.r.t $\{\widetilde{\mathcal{T}}_h, \widetilde{\mathcal{M}}_h\}$. The full conditionals of $\{\widetilde{\mathcal{T}}_h, \widetilde{\mathcal{M}}_h, \widetilde{\boldsymbol{\mathcal{M}}}_h^{(M)}\}_{h=1}^H$ are derived similarly, with the only difference being that the leaf node parameters are $(p_m+1)\times 1$ vectors due to the shared tree topologies. Assuming independence between $\widetilde{\boldsymbol{\mathcal{M}}}_h^{(M)}$ and $\widetilde{\mathcal{M}}_h$, we have

$$
\begin{aligned}
& p\left(\{\widetilde{\mathcal{T}}_h, \widetilde{\boldsymbol{\mathcal{M}}}_h^{(M)}, \widetilde{\mathcal{M}}_h\}_{h=1}^H | \mathcal{D}, \boldsymbol{\Sigma}, \{(\mathcal{T}_j, \boldsymbol{\mathcal{M}}_j)\}_{j=1}^J, \{\breve{\mathcal{T}}_k, \breve{\mathcal{M}}_k\}_{k=1}^K, \boldsymbol{\gamma}\right) \\
&= \prod_{h=1}^H p\left(\widetilde{\boldsymbol{\mathcal{M}}}_h^{(M)}, \widetilde{\mathcal{M}}_h | \widetilde{\boldsymbol{R}}_h^{(M)}, \widetilde{\boldsymbol{R}}_h, \widetilde{\mathcal{T}}_h, \boldsymbol{\gamma}, \boldsymbol{\Sigma}\right) p\left(\widetilde{\mathcal{T}}_h | \widetilde{\boldsymbol{R}}_h^{(M)}, \widetilde{\boldsymbol{R}}_h, \boldsymbol{\gamma}, \boldsymbol{\Sigma}\right) \\
&= \prod_{h=1}^H p\left(\widetilde{\mathcal{M}}_h | \widetilde{\boldsymbol{R}}_h, \widetilde{\mathcal{T}}_h, \boldsymbol{\gamma}\right) p\left(\widetilde{\boldsymbol{\mathcal{M}}}_h^{(M)} | \widetilde{\boldsymbol{R}}_h^{(M)}, \widetilde{\mathcal{T}}_h, \boldsymbol{\Sigma}\right) p\left(\widetilde{\mathcal{T}}_h | \widetilde{\boldsymbol{R}}_h^{(M)}, \widetilde{\boldsymbol{R}}_h, \boldsymbol{\gamma}, \boldsymbol{\Sigma}\right)
\end{aligned}
$$

where

$$
\begin{aligned}
p\left(\widetilde{\mathcal{M}}_h | \widetilde{\boldsymbol{R}}_h, \widetilde{\mathcal{T}}_h, \boldsymbol{\gamma}\right) &\propto p\left(\widetilde{\boldsymbol{R}}_h | \widetilde{\mathcal{M}}_h, \widetilde{\mathcal{T}}_h, \boldsymbol{\gamma}\right) p\left(\widetilde{\mathcal{M}}_h | \widetilde{\mathcal{T}}_h\right) \\
&\propto \prod_{l=1}^{b_h} \prod_{i:\mathbb{1}_{il}=1} \exp\left[\delta_i \left\{\widetilde{R}_{ih} + \widetilde{\mu}_{hl} A_i\right\} - \exp\left\{\widetilde{R}_{ih} + \widetilde{\mu}_{hl} A_i\right\} \int_0^{Y_i} \exp\left(\boldsymbol{\gamma}^T \mathbf{B}(t)\right) dt\right] \exp\left[\widetilde{\mu}_{hl}\widetilde{\zeta} - \exp(\widetilde{\mu}_{hl})\widetilde{\eta}\right] \\
&\propto \prod_{l=1}^{b_h} \exp\left[\widetilde{\mu}_{hl}\left(\sum_{i:\mathbb{1}_{il}=1} \delta_i A_i + \widetilde{\zeta}\right) - \exp(\widetilde{\mu}_{hl}) \left\{\widetilde{\eta} + \sum_{i:\mathbb{1}_{il}=1, A_i=1} \exp(\widetilde{R}_{ih}) \int_0^{Y_i} \exp\left(\boldsymbol{\gamma}^T \mathbf{B}(t)\right) dt\right\}\right] \\
&\stackrel{d}{=} \prod_{l=1}^{b_h} logGam(\widetilde{\zeta}^*, \widetilde{\eta}^*),
\end{aligned}
\tag{S.8}
$$

with $\widetilde{\zeta}^* = \sum_{i:\mathbb{1}_{il}=1} \delta_i A_i + \widetilde{\zeta}$ and $\widetilde{\eta}^* = \widetilde{\eta} + \sum_{i:\mathbb{1}_{il}=1, A_i=1} \exp(\widetilde{R}_{ih}) \int_0^{Y_i} \exp\left(\boldsymbol{\gamma}^T \mathbf{B}(t)\right) dt$; and

$$
\begin{aligned}
p\left(\widetilde{\boldsymbol{\mathcal{M}}}_h^{(M)} | \widetilde{\boldsymbol{R}}_h^{(M)}, \widetilde{\mathcal{T}}_h, \boldsymbol{\Sigma}\right) &\propto p\left(\widetilde{\boldsymbol{R}}_h^{(M)} | \widetilde{\boldsymbol{\mathcal{M}}}_h^{(M)}, \widetilde{\mathcal{T}}_h, \boldsymbol{\Sigma}\right) p\left(\widetilde{\boldsymbol{\mathcal{M}}}_h^{(M)} | \widetilde{\mathcal{T}}_h\right) \\
&\propto \prod_{l=1}^{b_h} \prod_{i:\mathbb{1}_{il}=1} \exp\left\{-\frac{1}{2}\left(\widetilde{\boldsymbol{R}}_{ih}^{(M)} - \widetilde{\boldsymbol{\mu}}_{hl}^{(M)}\right)^T \boldsymbol{\Sigma}^{-1} \left(\widetilde{\boldsymbol{R}}_{ih}^{(M)} - \widetilde{\boldsymbol{\mu}}_{hl}^{(M)}\right)\right\} \exp\left\{-\frac{1}{2}(\widetilde{\boldsymbol{\mu}}_{hl}^{(M)})^T \boldsymbol{\Sigma}_{\widetilde{\mu}}^{-1} \widetilde{\boldsymbol{\mu}}_{hl}^{(M)}\right\} \\
&\stackrel{d}{=} \prod_{l=1}^{b_h} N(\widetilde{\boldsymbol{a}}_l, \widetilde{\boldsymbol{\Sigma}}_l),
\end{aligned}
\tag{S.9}
$$

with $\widetilde{\boldsymbol{\Sigma}}_l = (\widetilde{n}_l \boldsymbol{\Sigma}^{-1} + \boldsymbol{\Sigma}_{\widetilde{\mu}}^{-1})^{-1}$, $\widetilde{\boldsymbol{a}}_l = \sum_{i:\mathbb{1}_{il}=1} (\widetilde{\boldsymbol{R}}_{ih}^{(M)})^T \boldsymbol{\Sigma}^{-1} (\widetilde{n}_l \boldsymbol{\Sigma}^{-1} + \boldsymbol{\Sigma}_{\widetilde{\mu}}^{-1})^{-1}$, and $\boldsymbol{\Sigma}_{\widetilde{\mu}} = \frac{c_2^2}{4Hk_2^2}\widetilde{\boldsymbol{\Sigma}}_0$.

Using the conjugate normal and log-gamma priors once again leads to closed-form marginal

posteriors for $\widetilde{\mathcal{T}}_h$, which is integrated over $\{\widetilde{\mathcal{M}}_h, \widetilde{\boldsymbol{\mathcal{M}}}_h^{(M)}\}$ and expressed as

$$
\begin{aligned}
p\left(\widetilde{\mathcal{T}}_h|\widetilde{\boldsymbol{R}}_h, \widetilde{\boldsymbol{R}}_h^{(M)}, \boldsymbol{\gamma}, \sigma\right) &\propto p(\widetilde{\mathcal{T}}_h) \int p\left(\widetilde{\boldsymbol{R}}_h|\widetilde{\mathcal{M}}_h, \widetilde{\mathcal{T}}_h, \boldsymbol{\gamma}\right) p\left(\widetilde{\mathcal{M}}_h|\widetilde{\mathcal{T}}_h\right) d\widetilde{\mathcal{M}}_h \int p\left(\widetilde{\boldsymbol{R}}_h^{(M)}|\widetilde{\boldsymbol{\mathcal{M}}}_h^{(M)}, \widetilde{\mathcal{T}}_h, \boldsymbol{\Sigma}\right) p\left(\widetilde{\boldsymbol{\mathcal{M}}}_h^{(M)}|\widetilde{\mathcal{T}}_h\right) d\widetilde{\boldsymbol{\mathcal{M}}}_h^{(M)} \\
&\propto p(\widetilde{\mathcal{T}}_h) \prod_{l=1}^{b_h} \int \exp\left[\sum_{i:\mathbb{1}_{il}=1} \delta_i \widetilde{R}_{ih} + \widetilde{\mu}_{hl} \sum_{i:\mathbb{1}_{il}=1} \delta_i A_i - \exp(\widetilde{\mu}_{hl}) \sum_{i:\mathbb{1}_{il}=A_i=1} \exp(\widetilde{R}_{ih}) \int_0^{Y_i} \exp\left(\boldsymbol{\gamma}^T \mathbf{B}(t)\right) dt \right. \\
&\left. - \sum_{i:\mathbb{1}_{il}=1, A_i=0} \exp(\widetilde{R}_{ih}) \int_0^{Y_i} \exp\left(\boldsymbol{\gamma}^T \mathbf{B}(t)\right) dt \right] \times \frac{\widetilde{\eta}^{\widetilde{\zeta}}}{\Gamma(\widetilde{\zeta})} \exp\left(\widetilde{\mu}_{hl}\widetilde{\zeta} - \exp(\widetilde{\mu}_{hl})\widetilde{\eta}\right) d\widetilde{\mu}_{hl} \\
&\times \int \prod_{i:\mathbb{1}_{il}=1} \frac{1}{\exp} \left\{-\frac{1}{2}\left(\widetilde{\boldsymbol{R}}_{ih}^{(M)} - \widetilde{\boldsymbol{\mu}}_{hl}^{(M)}\right)^T \boldsymbol{\Sigma}^{-1} \left(\widetilde{\boldsymbol{R}}_{ih}^{(M)} - \widetilde{\boldsymbol{\mu}}_{hl}^{(M)}\right)\right\} \exp\left\{-\frac{1}{2}(\widetilde{\boldsymbol{\mu}}_{hl}^{(M)})^T \boldsymbol{\Sigma}_{\widetilde{\mu}}^{-1} \widetilde{\boldsymbol{\mu}}_{hl}^{(M)}\right\} d\widetilde{\boldsymbol{\mu}}_{hl}^{(M)} \\
&\propto p(\widetilde{\mathcal{T}}_h) \prod_{l=1}^{b_h} \exp\left(\sum_{i:\mathbb{1}_{il}=1} \delta_i \widetilde{R}_{ih} - \sum_{i:\mathbb{1}_{il}=1, A_i=0} \exp(\widetilde{R}_{ih}) \int_0^{Y_i} \exp\left(\boldsymbol{\gamma}^T \mathbf{B}(t)\right) dt\right) \frac{\widetilde{\eta}^{\widetilde{\zeta}} \Gamma(\widetilde{\zeta}^*)}{\Gamma(\widetilde{\zeta})(\widetilde{\eta}^*)^{\widetilde{\zeta}^*}} \\
&\times \left(\frac{1}{\sqrt{2\pi}}\right)^{n_l p_m} \det(\boldsymbol{\Sigma})^{(-n_l/2)} \det(\boldsymbol{\Sigma}_\mu)^{(-1/2)} \det\left(\widetilde{\boldsymbol{\Sigma}}_l\right)^{1/2} \exp\left\{-\frac{1}{2} \sum_{i:\mathbb{1}_{il}=1} (\widetilde{\boldsymbol{R}}_{ih}^{(M)})^T \boldsymbol{\Sigma}^{-1} \widetilde{\boldsymbol{R}}_{ih}^{(M)}\right\} \\
&\times \exp\left\{\frac{1}{2}\left(\sum_{i:\mathbb{1}_{il}=1} \widetilde{\boldsymbol{R}}_{ih}^{(M)}\right)^T \boldsymbol{\Sigma}^{-1} \widetilde{\boldsymbol{\Sigma}}_l \boldsymbol{\Sigma}^{-1} \left(\sum_{i:\mathbb{1}_{il}=1} \widetilde{\boldsymbol{R}}_{ih}^{(M)}\right)\right\}. \\
&\propto p(\widetilde{\mathcal{T}}_h) \prod_{l=1}^{b_h} \det(\boldsymbol{\Sigma}_\mu)^{(-1/2)} \det\left(\widetilde{\boldsymbol{\Sigma}}_l\right)^{1/2} \exp\left\{-\frac{1}{2} \sum_{i:\mathbb{1}_{il}=1} (\widetilde{\boldsymbol{R}}_{ih}^{(M)})^T \boldsymbol{\Sigma}^{-1} \widetilde{\boldsymbol{R}}_{ih}^{(M)}\right\} \\
&\times \exp\left\{\frac{1}{2}\left(\sum_{i:\mathbb{1}_{il}=1} \widetilde{\boldsymbol{R}}_{ih}^{(M)}\right)^T \boldsymbol{\Sigma}^{-1} \widetilde{\boldsymbol{\Sigma}}_l \boldsymbol{\Sigma}^{-1} \left(\sum_{i:\mathbb{1}_{il}=1} \widetilde{\boldsymbol{R}}_{ih}^{(M)}\right)\right\}.
\end{aligned}
\tag{S.10}
$$

Posterior draws of $\widetilde{\mathcal{T}}_h$ can thus be obtained using MH algorithm with the same proposal distribution described above.

(III) **Full conditional distribution of** $\{\breve{\mathcal{T}}_k, \breve{\mathcal{M}}_k\}_{k=1}^K$

Let $\breve{R}_{ik} = \sum_{l \neq k} g_3(\mathbf{x}_i, M_i; \breve{\mathcal{T}}_l, \breve{\mathcal{M}}_l) + \tau(\mathbf{x}_i)A_i$ and $\breve{\boldsymbol{R}}_k = (\breve{R}_{1k}, \ldots, \breve{R}_{nk})^T$ denote the partial residuals in the PH model w.r.t $\{\breve{\mathcal{T}}_k, \breve{\mathcal{M}}_k\}$. The full conditionals of $\{\breve{\mathcal{T}}_k, \breve{\mathcal{M}}_k\}_{k=1}^K$ are derived as

$$
p\left(\{\breve{\mathcal{T}}_k, \breve{\mathcal{M}}_k\}_{k=1}^K|\mathcal{D}, \boldsymbol{\gamma}, \{\widetilde{\mathcal{T}}_h, \widetilde{\mathcal{M}}_h\}_{h=1}^H\right) = \prod_{k=1}^K p\left(\breve{\mathcal{M}}_k|\breve{\boldsymbol{R}}_k, \breve{\mathcal{T}}_k, \boldsymbol{\gamma}\right) p\left(\breve{\mathcal{T}}_k|\breve{\boldsymbol{R}}_k, \boldsymbol{\gamma}\right),
$$

where

$$
\begin{aligned}
p\left(\breve{\mathcal{M}}_k|\breve{\boldsymbol{R}}_k,\breve{\mathcal{T}}_k,\boldsymbol{\gamma}\right) &\propto p\left(\breve{\boldsymbol{R}}_k|\breve{\mathcal{M}}_k,\breve{\mathcal{T}}_k,\boldsymbol{\gamma}\right) p\left(\breve{\mathcal{M}}_k|\breve{\mathcal{T}}_k\right)\\
&\propto \prod_{l=1}^{b_k}\prod_{i:\mathbb{1}_{il}=1}\exp\left[\delta_i\left\{\breve{R}_{ik}+\breve{\mu}_{kl}\right\}-\exp\left\{\breve{R}_{ik}+\breve{\mu}_{kl}\right\}\int_0^{Y_i}\exp\left(\boldsymbol{\gamma}^T\mathbf{B}(t)\right)dt\right]\exp\left[\breve{\mu}_{kl}\breve{\zeta}-\exp(\breve{\mu}_{kl})\breve{\eta}\right]\\
&\propto \prod_{l=1}^{b_k}\exp\left[\breve{\mu}_{kl}\left(\sum_{i:\mathbb{1}_{il}=1}\delta_i+\breve{\zeta}\right)-\exp(\breve{\mu}_{kl})\left\{\breve{\eta}+\sum_{i:\mathbb{1}_{il}=1}\exp(\breve{R}_{ik})\int_0^{Y_i}\exp\left(\boldsymbol{\gamma}^T\mathbf{B}(t)\right)dt\right\}\right]\\
&\overset{d}{=}\prod_{l=1}^{b_k}\text{log-gamma}(\breve{\zeta}^*,\breve{\eta}^*),
\end{aligned}
\tag{S.11}
$$

with $\breve{\zeta}^*=\sum_{i:\mathbb{1}_{il}=1}\delta_i+\breve{\zeta}$ and $\breve{\eta}^*=\breve{\eta}+\sum_{i:\mathbb{1}_{il}=1}\exp(\breve{R}_{ik})\int_0^{Y_i}\exp\left(\boldsymbol{\gamma}^T\mathbf{B}(t)\right)dt$. The marginal posterior distribution of $\breve{\mathcal{T}}_k$ is then derived as

$$
\begin{aligned}
p(\breve{\mathcal{T}}_k|\breve{\boldsymbol{R}}_k,\boldsymbol{\gamma}) &\propto p(\breve{\mathcal{T}}_k)\int p\left(\breve{\boldsymbol{R}}_k|\breve{\mathcal{M}}_k,\breve{\mathcal{T}}_k,\boldsymbol{\gamma}\right)p\left(\breve{\mathcal{M}}_k|\breve{\mathcal{T}}_k\right)d\breve{\mathcal{M}}_k,\\
&\propto p(\breve{\mathcal{T}}_k)\prod_{l=1}^{b_k}\int\exp\left[\sum_{i:\mathbb{1}_{il}=1}\delta_i\breve{R}_{ik}+\breve{\mu}_{kl}\sum_{i:\mathbb{1}_{il}=1}\delta_i-\exp(\breve{\mu}_{kl})\sum_{i:\mathbb{1}_{il}=1}\exp(\breve{R}_{ik})\int_0^{Y_i}\exp\left(\boldsymbol{\gamma}^T\mathbf{B}(t)\right)dt\right]\\
&\times\frac{\breve{\eta}^{\breve{\zeta}}}{\Gamma(\breve{\zeta})}\exp\left\{\breve{\mu}_{kl}\breve{\zeta}-\exp(\breve{\mu}_{kl})\breve{\eta}\right\}d\breve{\mu}_{kl}\\
&\propto p(\breve{\mathcal{T}}_k)\prod_{l=1}^{b_k}\exp\left(\sum_{i:\mathbb{1}_{il}=1}\delta_i\breve{R}_{ik}\right)\int\frac{\breve{\eta}^{\breve{\zeta}}}{\Gamma(\breve{\zeta})}\exp\left[\breve{\mu}_{kl}\Big(\sum_{i:\mathbb{1}_{il}=1}\delta_i+\breve{\zeta}\Big)-\exp(\breve{\mu}_{kl})\Big\{\breve{\eta}\right.\\
&\left.+\sum_{i:\mathbb{1}_{il}=1}\exp(\breve{R}_{ik})\int_0^{Y_i}\exp\left(\boldsymbol{\gamma}^T\mathbf{B}(t)\right)dt\Big\}\right]d\breve{\mu}_{kl}\\
&\propto p(\breve{\mathcal{T}}_k)\prod_{l=1}^{b_k}\exp\left(\sum_{i:\mathbb{1}_{il}=1}\delta_i\breve{R}_{ik}\right)\frac{\breve{\eta}^{\breve{\zeta}}\Gamma(\breve{\zeta}^*)}{\Gamma(\breve{\zeta})(\breve{\eta}^*)^{\breve{\zeta}^*}}.
\end{aligned}
\tag{S.12}
$$

Web Appendix C Additional simulation results

C.1 Simulation with a fake mediator M_3

The estimation results based on 100 replications are summarized in Table S1. Figure S2 depicts the distribution of the true and estimated ICPSEs based on a randomly selected replication. Figure S3 shows the average posterior splitting proportions of the predictors.

C.2 Sensitivity analyses

We investigated the robustness of the proposed method to different choices of hyperparameters, the number of spline basis functions, baseline hazard, and censoring rate. We used setup (i) with a sample size of $n = 1,000$ to present the results. Seven scenarios were considered as follows: (I) $\alpha = \breve{\alpha} = 0.95, \widetilde{\alpha} = 0.25$; $\beta = \breve{\beta} = 2, \widetilde{\beta} = 3$; (II) $\alpha = \breve{\alpha} = \widetilde{\alpha} = 0.95$; $\beta = \breve{\beta} = \widetilde{\beta} = 2$; (III) $J = K = 200$, $H = 50$; (IV) $J = K = H = 100$; (V) $L = 20$; (VI) $\lambda_0(t) = 2\mathrm{t} + 2$; and (VII) $\lambda_0(t) = 1, CR = 40\%$. Note that scenario (I) constrained each binary tree $\widetilde{\mathcal{T}}_h$ to even shallower depths compared to the default prior through the decreased $\widetilde{\alpha}$, while scenario (II) allows each $\widetilde{\mathcal{T}}_h$ to grow deeper than those under the default prior through the decrease $\widetilde{\beta}$. Table S2 presents the estimation results under each scenario based on 100 replications. Furthermore, to demonstrate the robustness of the proposed method with respective to violations of the normal assumption in the mediator regression model, we also considered non-normally distributed mediators under setup (i), where the residual terms in Equation (2) were generated from Student's t, Gamma, or

mixture Gaussian distributions as follows:

$$
\begin{aligned}
&(\mathrm{I}') : \epsilon_{i1} \overset{i.i.d}{\sim} t(3),\ \epsilon_{i2} \overset{i.i.d}{\sim} t(5);\\
&(\mathrm{II}') : \epsilon_{i1} \overset{i.i.d}{\sim} Gamma(3,2),\ \epsilon_{i2} \overset{i.i.d}{\sim} Gamma(3,2);\\
&(\mathrm{III}') : \boldsymbol{\epsilon_i} \overset{i.i.d}{\sim} \frac{2}{3}N\left(\begin{bmatrix}-0.5\\-0.5\end{bmatrix}, \begin{bmatrix}0.5 & -0.3\\-0.3 & 0.5\end{bmatrix}\right) + \frac{1}{3}N\left(\begin{bmatrix}1\\1\end{bmatrix}, \begin{bmatrix}0.5 & 0\\0 & 0.5\end{bmatrix}\right).
\end{aligned}
\tag{S.13}
$$

The estimation results based on 100 replications are summarized in Table S3.

The performance of the proposed method exhibits overall stability under varying strengths of prior regularization on the individual binary trees, while halving the number of trees in the ensembles leads to a slight increase in bias and RMSE for the estimated sample average interventional PSEs and $\sqrt{\text{PEHE}}$ for the ICPSEs as well. This behavior is reasonable, as shallow and few binary trees may overlook or miss important covariates. On the other hand, increasing the number of spline basis functions mildly improves the proposed model's performance. We have also considered another choice of $L = 5$ for the spline basis functions. The results obtained were found to be negligibly different from those presented in Table 1, and hence not reported for the sake of brevity. In scenarios with higher censoring proportions, a more noticeable increase in bias and RMSE is observed for the estimated sample average interventional for the estimated sample average interventional PSEs. This observation aligns with expectation, as higher levels of censoring generally lead to decreased estimation accuracy in survival models (see, e.g., Qin and Shen[10]; Kang et al.[11]). Finally, when the mediators are non-normally distributed, i.e., with residuals terms generated from heavy-tailed, skewed, or mixture distributions, the results presented in Table S3 indicate robustness of the proposed method. Only a slight increase is observed in the bias, RMSE, and $\sqrt{\text{PEHE}}$ of the estimated causal effects.

C.3 Additional simulation on variable selection performance

To further evaluate the variable selection performance of the proposed method, we considered two additional scenarios, referred to as setups (iii) and (iv), where the number of pre-treatment covariates or the number of candidate mediators increased with the number of observations. Both setups were conceptualized based on setup (i) of the simulation study to facilitate a fair comparison with the results obtained in Table 1. Specifically, setup (iii) corresponds to a case where the dimension of pre-treatment covariates, p, increased from 25 to 50 as the number of observations, n, doubled, while the dimensions of the candidate mediators and true confounders, as well as their function in the data generating process, remain unchanged from setup (i). Building upon setup (iii), setup (iv) further accounts for a situation where the number of candidate mediators, p_m, also doubled with p and n (i.e., $n = 2,000$, $p = 50$, $p_m = 4$) . Given that genuine mediators are intrinsically rare to discover in practical applications, we generated two fake mediators, denoted by

$$\begin{aligned} &M_3 \sim N\Big(-0.5x_1 + 0.5\sin(\pi(x_9 - 1)) + 0.5(|x_2 + x_6| - 1)A, 1\Big) \\ &M_4 \sim N\big(0.5(x_4 + x_3), 1\big), \end{aligned} \tag{S.14}$$

apart from the real ones in setup (i), with a modified mediator-outcome relationship,

$$\upsilon^*(\mathbf{x}_i, \mathbf{M}_i) = -0.2|x_{i3}| + 0.5x_{i,12} + M_{i1} + \big(1 + 0.5I(x_{i6} > 0.5)\big)M_{i2} - 0.5|M_{i4}|,$$

considered in the PH model. In other words, M_3 was designed to be affected by the treatment but have no effect on the survival outcome, and M_4 vice versa. The remaining conditions were all the same as in setup (i). Under each setup, we generated 100 replicated datasets to conduct analysis. The corresponding results are summarized in Table S4.

By comparing with the left panel of Table 1, we noticed that the proposed method

performed stably in terms of bias, RMSE, and $\sqrt{\text{PEHE}}$ of the estimated effects when the dimension of pre-treatment covariates, p, and the number of observations, n, were both doubled. Additionally, by comparing with the right panel of Table 1, we found that increasing the number of redundant covariates with the sample size fixed at $n = 2,000$ only led to a slightly increase in bias, RMSE, and $\sqrt{\text{PEHE}}$. Similar results were obtained when p_m also increased with p and n. Figure S6- S7 shows the average posterior splitting proportions of the predictors in each tree ensemble based on the 100 replications. Despite the increasing number of irrelevant covariates, the proposed method selected the true medaitors, confounders, and effect modifiers with high probabilities, while excluding the redundant ones with near-zero selection probabilities.

Table S1: Average bias, relative bias, RMSE, coverage rate of the 95% credible interval for the sample average interventional PSEs, and $\sqrt{\text{PEHE}}$ for the ICPSEs on the scale of logarithm of hazards and survival probability at the mean observed event time under setup (ii) with a fake M_3.

		$n = 1,000$				$n = 2,000$			
PSE	Criterion	Gibbs type		Discrete Uniform		Gibbs type		Discrete Uniform	
		Logh	Survp	Logh	Survp	Logh	Survp	Logh	Survp
DE	Bias	0.086	−0.007	0.245	−0.034	0.057	−0.005	0.146	−0.016
	RBias	0.116	0.097	0.291	0.272	0.080	0.064	0.197	0.159
	RMSE	0.120	0.013	0.269	0.034	0.086	0.010	0.156	0.029
	$\sqrt{\text{PEHE}}$	0.393	0.063	0.520	0.084	0.314	0.057	0.405	0.072
IE_1	Bias	−0.113	0.018	−0.275	0.038	−0.079	0.013	−0.168	0.030
	RBias	0.089	0.113	0.209	0.242	0.060	0.072	0.134	0.152
	RMSE	0.144	0.019	0.309	0.042	0.098	0.016	0.199	0.032
	$\sqrt{\text{PEHE}}$	0.445	0.066	0.615	0.079	0.317	0.055	0.449	0.068
IE_2	Bias	−0.046	0.011	−0.069	0.014	−0.029	0.006	−0.047	0.010
	RBias	0.103	0.133	0.122	0.144	0.074	0.086	0.091	0.112
	RMSE	0.089	0.014	0.095	0.017	0.070	0.011	0.083	0.014
	$\sqrt{\text{PEHE}}$	0.614	0.069	0.646	0.074	0.463	0.057	0.539	0.066
IE_3	Bias	0	0	0.009	−0.001	0	0	0.008	−0.001
	RBias	-	-	-	-	-	-	-	-
	RMSE	2e−4	0	0.043	0.004	1e−4	0	0.027	0.003
	$\sqrt{\text{PEHE}}$	0	0	0.059	0.010	0	0	0.041	0
IE_J	Bias	−0.161	0.024	−0.360	0.050	−0.106	0.016	−0.225	0.033
	RBias	0.088	0.097	0.174	0.183	0.053	0.056	0.123	0.114
	RMSE	0.223	0.028	0.419	0.057	0.142	0.019	0.288	0.039
	$\sqrt{\text{PEHE}}$	0.818	0.106	0.961	0.127	0.602	0.089	0.754	0.100
TE	Bias	−0.109	0.020	−0.122	0.021	−0.048	0.008	−0.087	0.007
	RBias	0.032	0.053	0.036	0.034	0.031	0.037	0.030	0.028
	RMSE	0.254	0.033	0.242	0.029	0.111	0.016	0.132	0.018
	$\sqrt{\text{PEHE}}$	0.890	0.117	0.944	0.125	0.687	0.094	0.762	0.108

$\star$ DE: the direct effect $A \to T$. IE_1 - IE_3: the separate indirect effect of M_1 - M_3.
IE_J: the joint indirect effect. TE: the total effect.
Logh: the logarithm of hazards. Survp: survival probability.

Table S2: Average bias, relative bias, RMSE, coverage rate of the 95% credible interval for the sample average interventional PSEs, and $\sqrt{\text{PEHE}}$ for the ICPSEs on the scale of logarithm of hazards and survival probability under scenarios (I) - (VII) with $n = 1,000$ and $p_m = 2$.

PSE	Crt.	(I)		(II)		(III)		(IV)		(V)		(VI)		(VII)	
		Logh	Survp	Logh	Survp	Logh	Survp	Logh	Survp	Logh	Survp	Logh	Survp	Logh	Survp
DE	Bias	0.093	−0.007	0.076	−0.004	0.078	−0.004	0.090	−0.008	0.082	−0.006	0.019	−0.003	0.113	−0.010
	RBias	0.121	0.099	0.118	0.082	0.113	0.080	0.123	0.097	0.113	0.086	0.079	0.086	0.150	0.114
	RMSE	0.127	0.014	0.113	0.012	0.116	0.012	0.126	0.015	0.115	0.011	0.082	0.013	0.152	0.015
	$\sqrt{\text{PEHE}}$.	0.467	0.072	0.377	0.060	0.391	0.062	0.398	0.065	0.389	0.061	0.388	0.060	0.471	0.061
IE_1	Bias	−0.086	0.012	−0.071	0.011	−0.075	0.011	−0.090	0.012	−0.092	0.015	−0.108	0.015	−0.093	0.014
	RBias	0.065	0.088	0.058	0.082	0.061	0.082	0.068	0.089	0.077	0.095	0.101	0.101	0.079	0.107
	RMSE	0.112	0.015	0.098	0.015	0.101	0.014	0.113	0.015	0.121	0.019	0.162	0.019	0.128	0.021
	$\sqrt{\text{PEHE}}$	0.344	0.053	0.400	0.058	0.356	0.054	0.435	0.057	0.404	0.059	0.408	0.061	0.410	0.067
IE_2	Bias	−0.036	0.007	−0.031	0.008	−0.033	0.007	−0.060	0.010	−0.040	0.010	−0.075	0.010	−0.066	0.009
	RBias	0.088	0.109	0.089	0.112	0.088	0.109	0.107	0.129	0.093	0.115	0.108	0.113	0.116	0.117
	RMSE	0.085	0.011	0.086	0.011	0.085	0.011	0.099	0.013	0.086	0.013	0.103	0.013	0.105	0.014
	$\sqrt{\text{PEHE}}$	0.532	0.061	0.556	0.064	0.524	0.061	0.558	0.065	0.557	0.063	0.552	0.067	0.555	0.075
IE_J	Bias	−0.123	0.016	−0.103	0.015	−0.108	0.015	−0.151	0.017	−0.113	0.016	−0.204	0.020	−0.157	0.022
	RBias	0.065	0.072	0.061	0.067	0.062	0.067	0.074	0.072	0.062	0.066	0.100	0.079	0.076	0.085
	RMSE	0.168	0.021	0.154	0.020	0.157	0.020	0.190	0.022	0.159	0.020	0.243	0.024	0.193	0.026
	$\sqrt{\text{PEHE}}$	0.667	0.087	0.734	0.095	0.674	0.088	0.745	0.095	0.730	0.094	0.741	0.098	0.740	0.102
TE	Bias	−0.030	0.009	−0.026	0.011	−0.030	0.011	−0.062	0.009	−0.050	0.010	−0.094	0.017	−0.038	0.010
	RBias	0.034	0.038	0.033	0.041	0.035	0.039	0.039	0.039	0.037	0.038	0.068	0.053	0.037	0.043
	RMSE	0.114	0.018	0.131	0.019	0.132	0.020	0.147	0.019	0.136	0.018	0.173	0.025	0.146	0.021
	$\sqrt{\text{PEHE}}$	0.776	0.106	0.817	0.110	0.762	0.105	0.829	0.110	0.820	0.109	0.826	0.111	0.852	0.114

Table S3: Average bias, relative bias, RMSE, coverage rate of the 95% credible interval for the sample average interventional PSEs, and $\sqrt{\text{PEHE}}$ for the ICPSEs on the scale of logarithm of hazards and survival probability under scenarios (I′) - (III′) with $n = 1,000$, $p_m = 2$, and non-normal residual terms.

PSE	Crt.	(I′)		(II′)		(III′)	
		Logh	Survp	Logh	Survp	Logh	Survp
DE	Bias	0.094	−0.010	0.090	−0.009	0.076	−0.004
	RBias	0.127	0.104	0.119	0.097	0.109	0.089
	RMSE	0.130	0.016	0.120	0.015	0.116	0.013
	$\sqrt{\text{PEHE}}$.	0.410	0.066	0.389	0.065	0.386	0.062
IE_1	Bias	−0.105	0.014	−0.119	0.017	−0.084	0.012
	RBias	0.086	0.100	0.092	0.117	0.080	0.097
	RMSE	0.139	0.018	0.150	0.021	0.120	0.017
	$\sqrt{\text{PEHE}}$	0.419	0.065	0.418	0.066	0.409	0.059
IE_2	Bias	−0.045	0.008	−0.049	0.010	−0.043	0.009
	RBias	0.101	0.117	0.100	0.126	0.092	0.121
	RMSE	0.093	0.012	0.089	0.013	0.087	0.012
	$\sqrt{\text{PEHE}}$	0.572	0.071	0.572	0.070	0.563	0.065
IE_J	Bias	−0.150	0.020	−0.167	0.024	−0.125	0.019
	RBias	0.079	0.083	0.081	0.090	0.071	0.080
	RMSE	0.196	0.025	0.199	0.026	0.180	0.024
	$\sqrt{\text{PEHE}}$	0.736	0.099	0.732	0.099	0.744	0.095
TE	Bias	−0.057	0.010	−0.077	0.015	−0.049	0.012
	RBias	0.045	0.041	0.040	0.045	0.042	0.043
	RMSE	0.160	0.020	0.145	0.020	0.156	0.019
	$\sqrt{\text{PEHE}}$	0.826	0.113	0.819	0.112	0.826	0.110

Table S4: Average bias, relative bias, RMSE, coverage rate of the 95% credible interval for the sample average interventional PSEs, and $\sqrt{\text{PEHE}}$ for the ICPSEs on the scale of logarithm of hazards and survival probability under setup (iii) - (iv) with $n = 2000$, $p = 50$, and $p_m = 2$ or 4.

PSE	Crt.	(iii)		(iv)	
		Logh	Survp	Logh	Survp
DE	Bias	-0.096	-0.009	0.078	-0.007
	RBias	0.119	0.085	0.098	0.081
	RMSE	0.114	0.012	0.105	0.012
	$\sqrt{\text{PEHE}}$.	0.325	0.052	0.350	0.056
IE_1	Bias	-0.110	0.013	-0.091	0.010
	RBias	0.079	0.092	0.092	0.102
	RMSE	0.123	0.015	0.115	0.013
	$\sqrt{\text{PEHE}}$	0.332	0.049	0.327	0.045
IE_2	Bias	-0.056	0.008	-0.054	0.008
	RBias	0.082	0.106	0.083	0.097
	RMSE	0.078	0.010	0.075	0.010
	$\sqrt{\text{PEHE}}$	0.474	0.055	0.499	0.060
IE_3	Bias	-	-	0	0
	RBias	-	-	-	-
	RMSE	-	-	0	0
	$\sqrt{\text{PEHE}}$	-	-	0	0
IE_4	Bias	-	-	0	0
	RBias	-	-	-	-
	RMSE	-	-	0.004	3e$-$4
	$\sqrt{\text{PEHE}}$	-	-	0.036	0.003
IE_J	Bias	-0.166	0.017	-0.134	0.015
	RBias	0.077	0.070	0.083	0.074
	RMSE	0.185	0.019	0.187	0.018
	$\sqrt{\text{PEHE}}$	0.625	0.079	0.628	0.079
TE	Bias	-0.070	0.009	-0.067	0.007
	RBias	0.033	0.032	0.038	0.035
	RMSE	0.120	0.014	0.126	0.014
	$\sqrt{\text{PEHE}}$	0.675	0.090	0.684	0.092

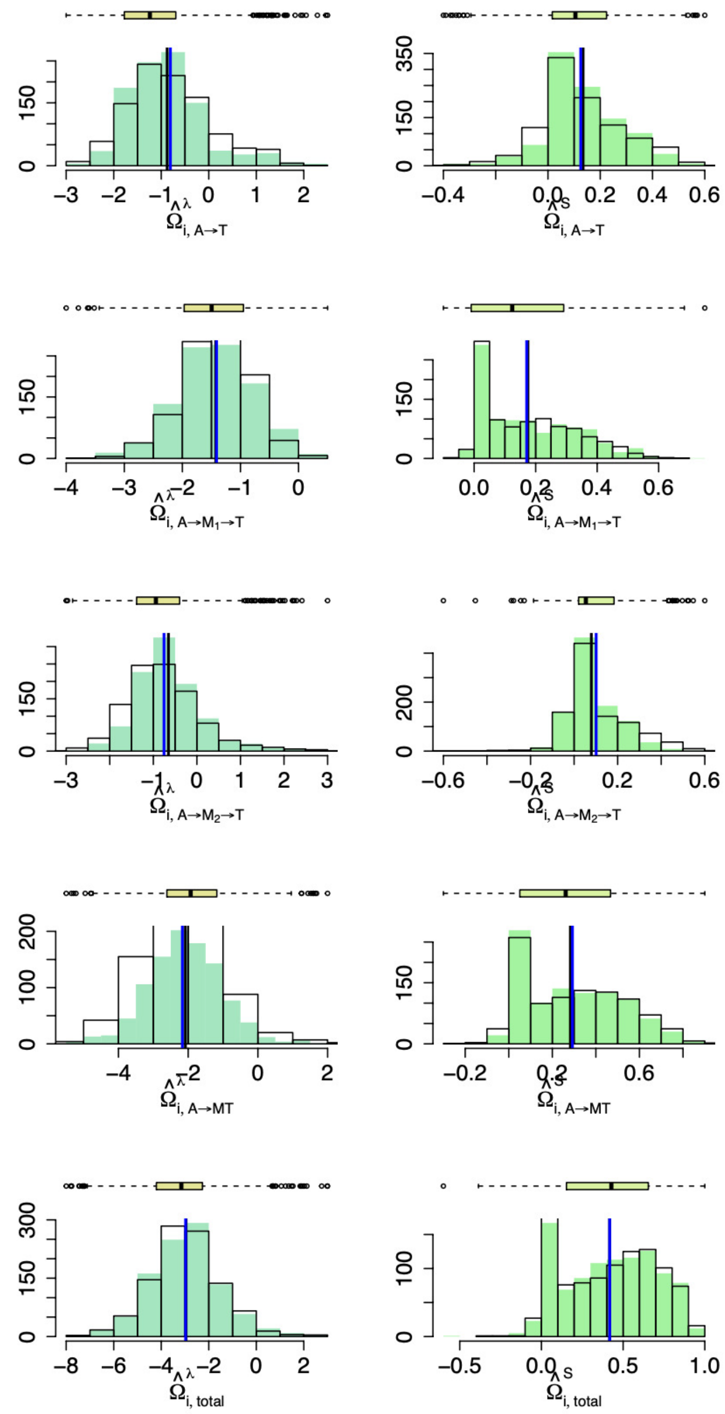

Figure S1: True and estimated ICPSEs for each individual on the logarithm scale of hazards (left) and survival probability with respect to mean observed survival time (right) based on a randomly selected dataset among the 100 replications under setup (i) with $p_m = 2$ mediators. The vertical blue and black lines stand for the true and estimated sample average interventional PSEs correspondingly.

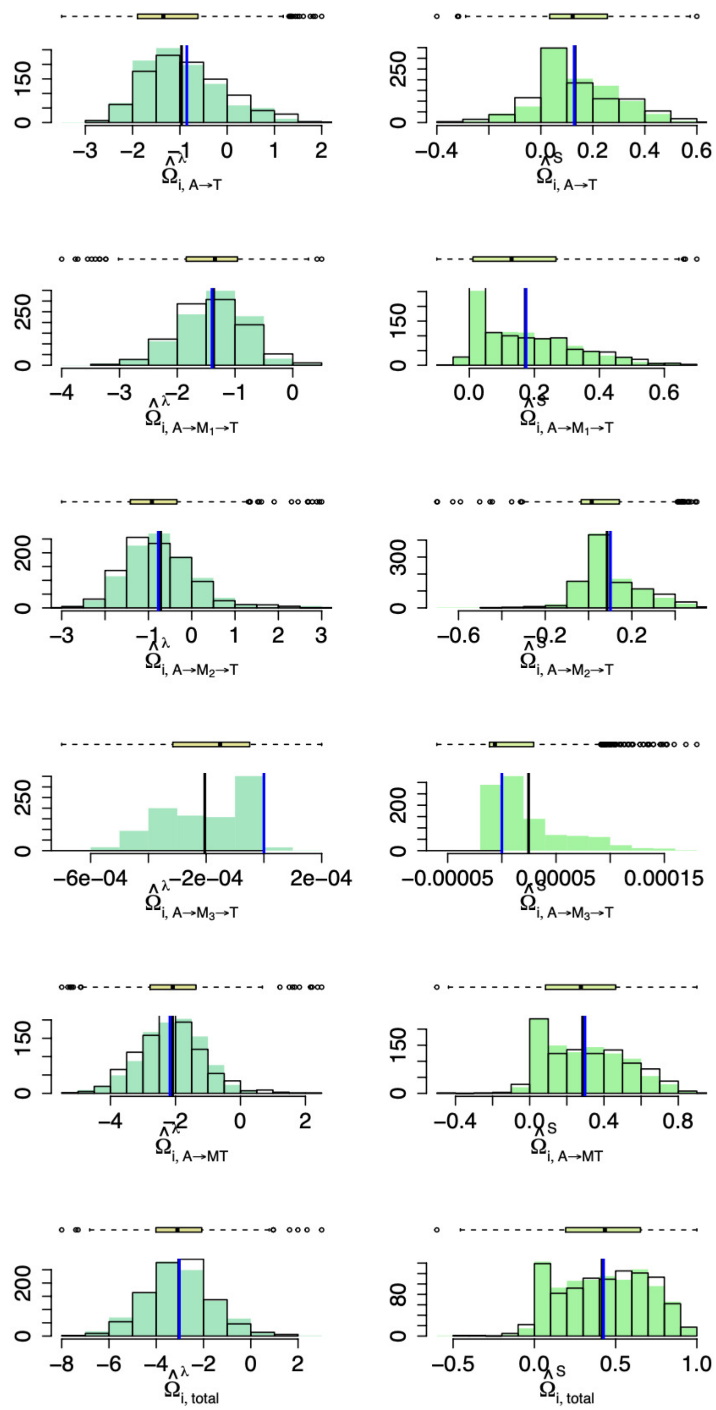

Figure S2: True and estimated ICPSEs for each individual on the logarithm scale of hazards (left) and survival probability with respect to mean observed survival time (right) based on a randomly selected dataset among the 100 replications under setup (ii) with a fake M_3.

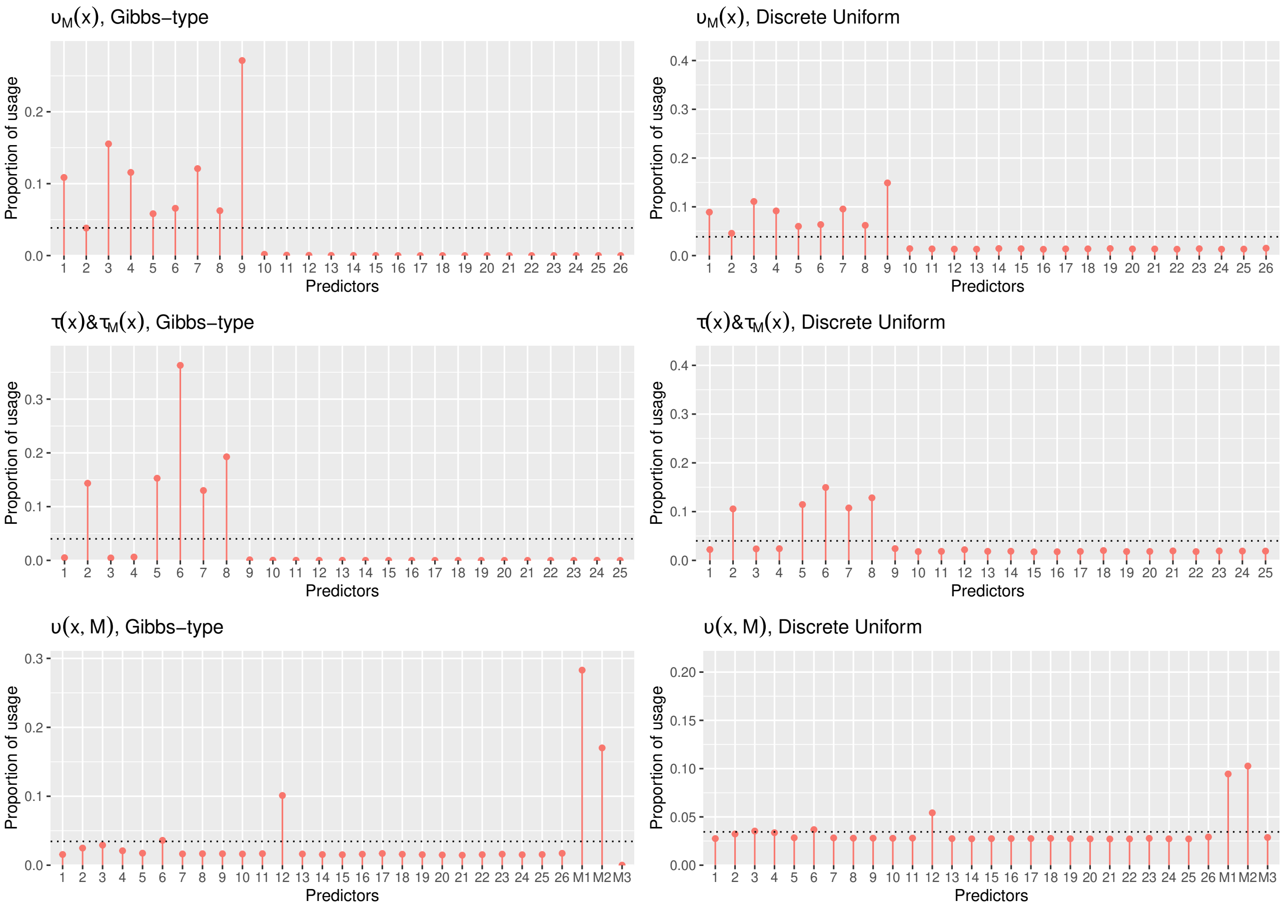

Figure S3: Posterior splitting proportions in the tree ensembles for each covariate under the Gibbs-type prior (left) and the default discrete uniform prior (right) in setup (ii) with $n = 1,000$. The horizontal dotted lines stand for the discrete uniform splitting probabilities.

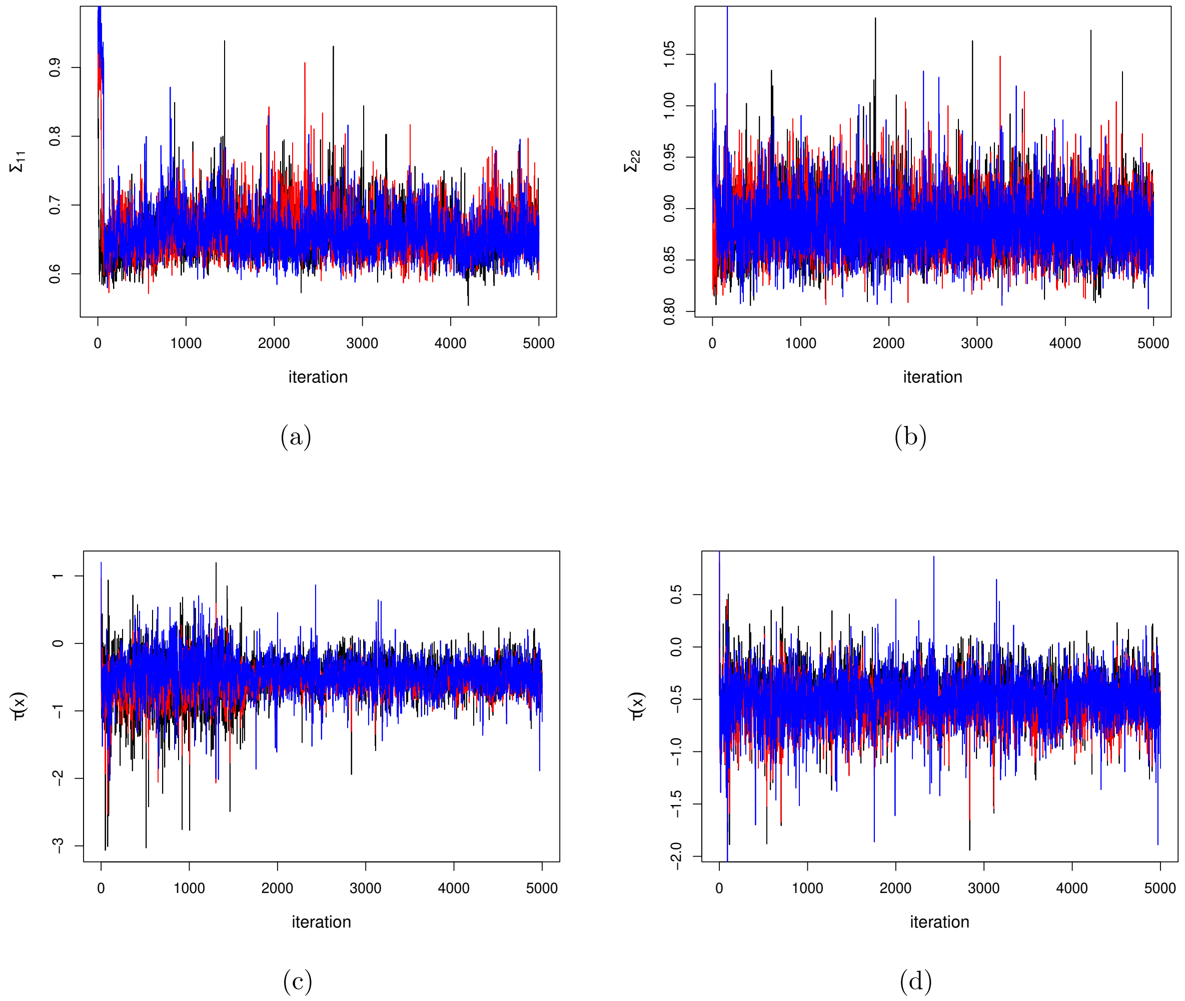

Figure S4: Trace plots of (a) Σ_{11}, (b) Σ_{22}, and (c)-(d) two randomly selected $\tau(\mathbf{x}_i)$ in the analysis of ACTG175 data.

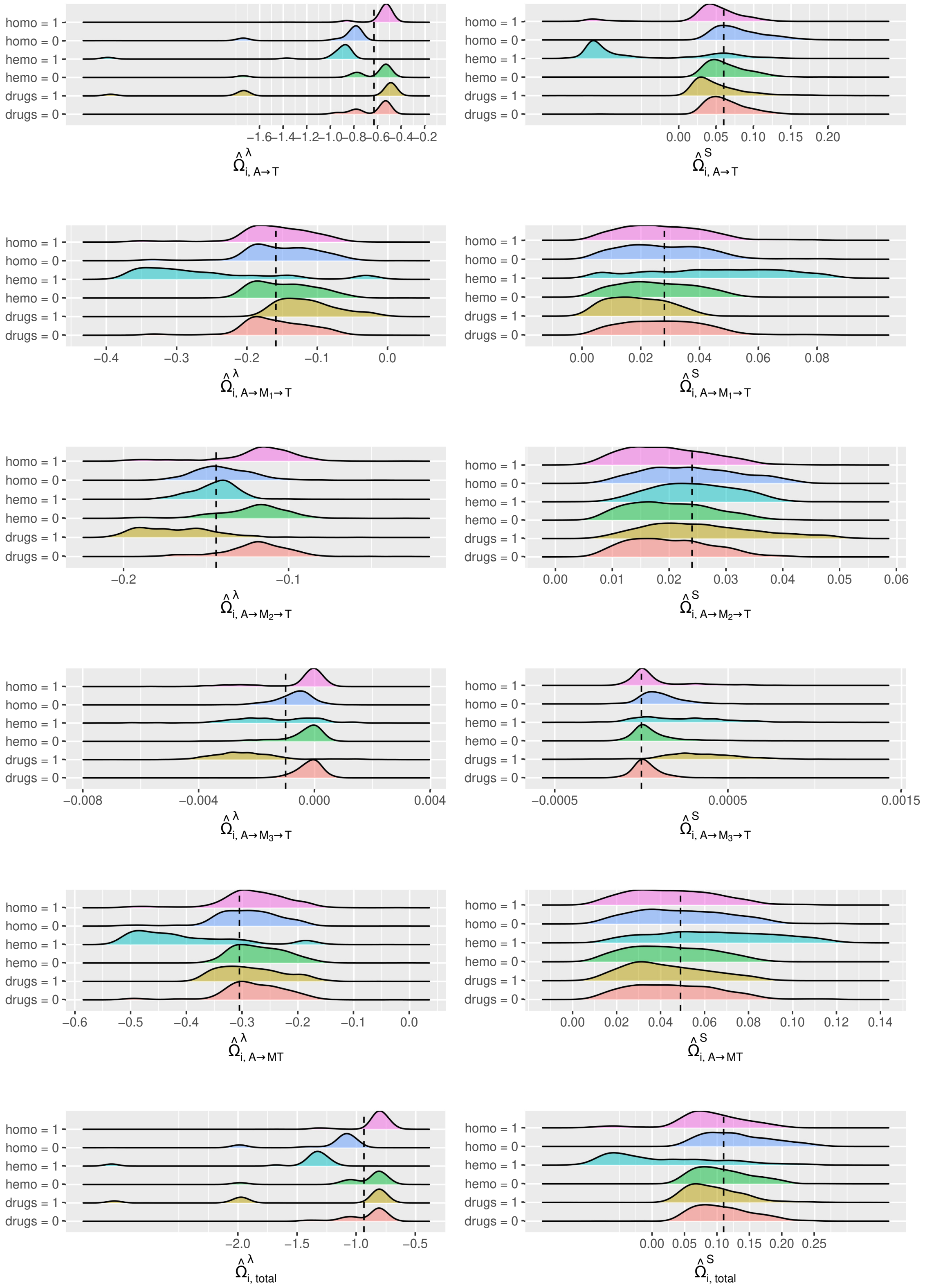

Figure S5: Partial effects of three effect modifiers: hemophilia, homosexual activity, and intravenous drug use history, on the distribution of the ICPSEs along each causal pathway. The dashed vertical lines represent the estimated sample average PSEs.

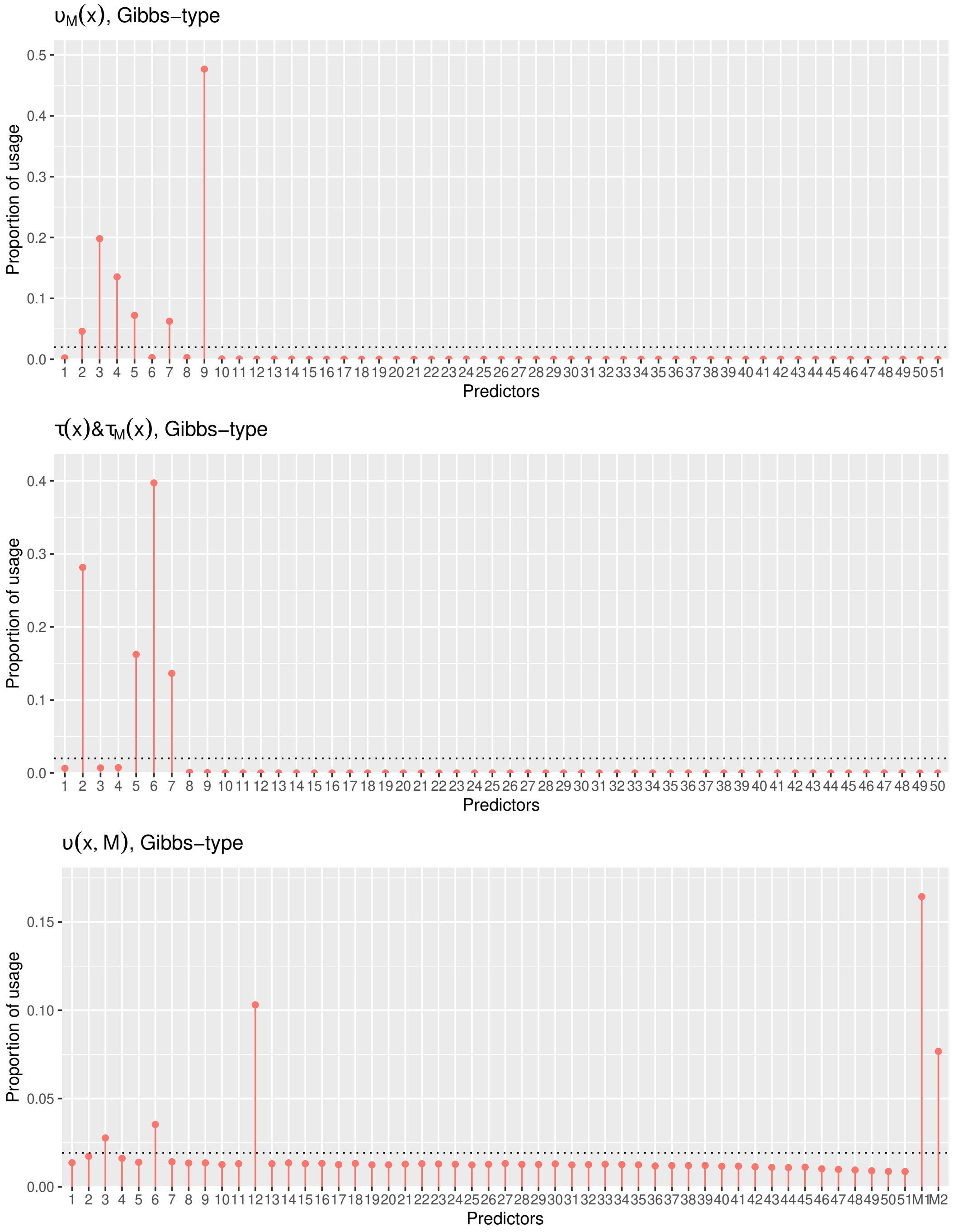

Figure S6: Posterior splitting proportions in the tree ensembles for each covariate, obtained by the proposed method under setup (iii) with $n = 2,000$, $p = 50$, $p_m = 2$. The horizontal dotted lines stand for the discrete uniform splitting probabilities.

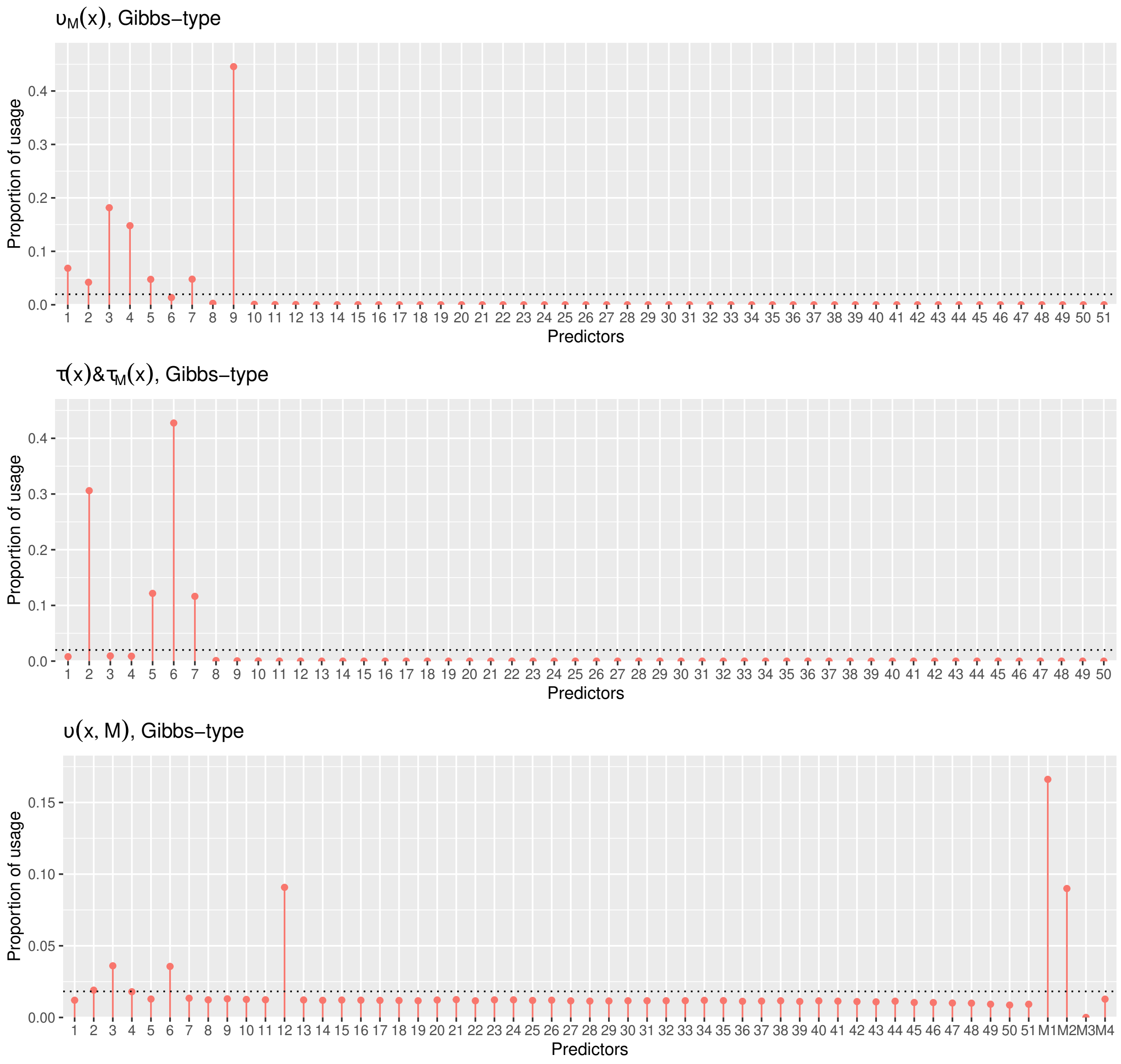

Figure S7: Posterior splitting proportions in the tree ensembles for each covariate, obtained by the proposed method under setup (iv) with $n = 2,000$, $p = 50$, $p_m = 4$. The horizontal dotted lines stand for the discrete uniform splitting probabilities.

References

1. Imai K, Keele L, Yamamoto T. Identification, Inference and Sensitivity Analysis for Causal Mediation Effects. *Stat Sci.* 2010;25(1):51–71.

2. Ten Have TR, Joffe MM. A review of causal estimation of effects in mediation analyses. *Stat Methods Med Res.* 2012;21(1):77–107.

3. Linero AR, Zhang Q. Mediation analysis using Bayesian tree ensembles. *Psychol Methods.* 2022.

4. Albert JM, Nelson S. Generalized causal mediation analysis. *Biometrics.* 2011;67(3):1028–1038.

5. Bonetti M, Gelber RD. Patterns of treatment effects in subsets of patients in clinical trials. *Biostatistics.* 2004;5(3):465–481.

6. Metropolis N, Rosenbluth AW, Rosenbluth MN, Teller AH, Teller E. Equation of State Calculations by Fast Computing Machines. *J Chem Phys.* 1953;21(6):1087–1092.

7. Hastings WK. Monte Carlo sampling methods using Markov chains and their applications. *Biometrika.* 1970;57(1):97–109.

8. Chipman HA, George EI, McCulloch RE. BART: Bayesian additive regression trees. *Ann Appl Stat.* 2010;4(1):266–298.

9. Linero AR, Du J. Gibbs Priors for Bayesian Nonparametric Variable Selection with Weak Learners. *J Comput Graph Stat.* 2023;32(3):1046–1059.

10. Qin J, Shen Y. Statistical Methods for Analyzing Right-Censored Length-Biased Data under Cox Model. *Biometrics.* 2010;66(2):382–392.

11. Kang K, Pan D, Song X. A joint model for multivariate longitudinal and survival data to discover the conversion to Alzheimer's disease. *Stat Med.* 2022;41(2):356–373.
